# Stab2-Mediated Clearance of Supramolecular Polymer Nanoparticles in Zebrafish Embryos

**DOI:** 10.1021/acs.biomac.9b01318

**Published:** 2020-02-21

**Authors:** Victorio Saez Talens, Gabriela Arias-Alpizar, D. M. M. Makurat, Joyal Davis, Jeroen Bussmann, Alexander Kros, Roxanne E. Kieltyka

**Affiliations:** Supramolecular and Biomaterials Chemistry, Leiden Institute of Chemistry, Leiden University, PO Box 9502, 2300 RA Leiden, The Netherlands

## Abstract

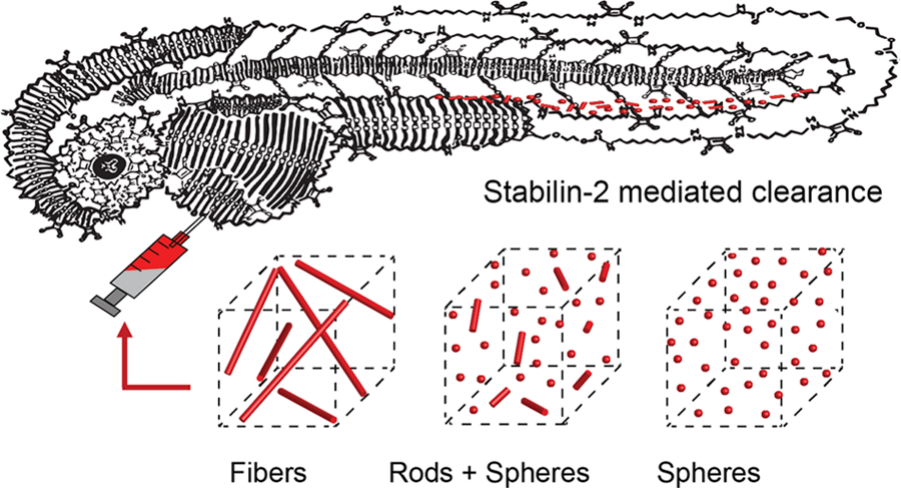

Supramolecular polymers are attractive scaffolds for use as nanocarriers in drug delivery thanks to their modularity and easy fabrication; however, a molecular view into their in vivo behavior is lacking. Herein, we prepare fluorescent squaramide-based supramolecular polymer nanoparticles that range from fibers to spheres while maintaining their surface chemistry and near-neutral surface charge by a co-assembly approach involving a sulfo-cyanine-labeled monomer to track their in vivo biodistribution behavior and clearance in optically transparent zebrafish embryos. Evasion of macrophages, localization of the fibrillar aggregates in the caudal vein, and association with scavenger endothelial cells are observed. The interaction of the fibrillar supramolecular nanoparticles with the caudal vein is abrogated in gene-edited zebrafish lacking Stabilin-2, a receptor analogously found in the mammalian liver, providing a molecular view into their interaction with scavenger endothelial cells. We further show that this interaction can be tuned based on the choice of monomer and its resultant self-assembly.

## Introduction

Significant effort has been dedicated to the design and synthesis of nanoparticle carriers (<200 nm in any dimension) with precisely engineered physicochemical properties for the targeted delivery of therapeutics. To this end, nanoparticles of varied features, including size, shape, surface charge, functionality, and elasticity, have been prepared.^1−4^ Irrespective of their designs, it has been demonstrated that >99% of such nanoparticles are cleared by the liver, yet the complete structure–activity relationships that underlie these processes remain unclear due to the inherent difficulty to visualize these cellular mechanisms in mammalian models.^2,5^ As a result, efficient in vivo targeting with minimal side effects is rarely achieved. This unsolved challenge highlights the urgent need to gain insight into (physico)chemical features that trigger the clearance of self-assembled particles by key organs at the molecular level to guide their rational design and administration in vivo.

A promising alternative to gain insight into the study of such particles in vivo at the cellular level is the zebrafish (*Danio rerio*) embryo.^6,7^ This organism provides unparalleled opportunities in comparison to first-line mouse models used to assess nanoparticle fate in vivo due to its optical transparency, small injection volumes, ease of genetic manipulation (i.e., CRISPR/cas), homology with 70% of human disease genes, fast development, and external fertilization.^8−13^ The high speed of the assays, low cost, and unprecedented (subcellular) resolution that can be obtained over the entire zebrafish embryo is leading to its rapid adoption within drug discovery pipelines as an efficient, accurate, and complementary model organism for in vivo prescreening of nanomedicines.^6^

Recently, we have demonstrated the functional homology of the zebrafish embryonic caudal vein (CV) to the mammalian liver at the molecular level, enabling the rapid screening of nanoparticle biodistribution and tissue targeting in vivo.^5^ Within the CV, two cell types dominate nanoparticle clearance: resident macrophages (similar to mammalian Kupffer cells) and specialized scavenger endothelial cells (SECs), which are functionally homologous to mammalian liver sinusoidal endothelial cells (LSECs). Mechanistically, we showed that the scavenger receptor Stabilin-2 dominates the endothelial clearance of isotropic, largely anionic nanoparticles. However, validating the scope of this receptor in mediating the clearance of nanocarriers with a wider range of physicochemical properties becomes critical to guide their in vivo application and to further benchmark the zebrafish embryo model in the field of drug delivery.

Significant differences in internalization, circulation times, stability, and cytotoxicity have been demonstrated with the modulation of nanoparticle shape, with contrasts often observed between spherical and fibrillar nanoparticles.^1,14−17^ Previously, in a primate model, anisotropic single-walled carbon nanotubes (SWCNTs) of length from 200 to 300 nm with a negative zeta potential have also been shown to be selectively cleared by LSECs.^18^ Moreover, in the same study, it was demonstrated that the Stabilin receptors mediate binding and endocytosis of SWCNTs in vitro. We, thus, became interested in the potential to further understand the biodistribution behavior in vivo of near-neutral, soft polymeric nanoparticles of distinct shapes ranging from fibers to spheres, focusing on their interaction with LSECs through the Stabilin-2 receptor in the zebrafish embryo model.

**Figure 1:**
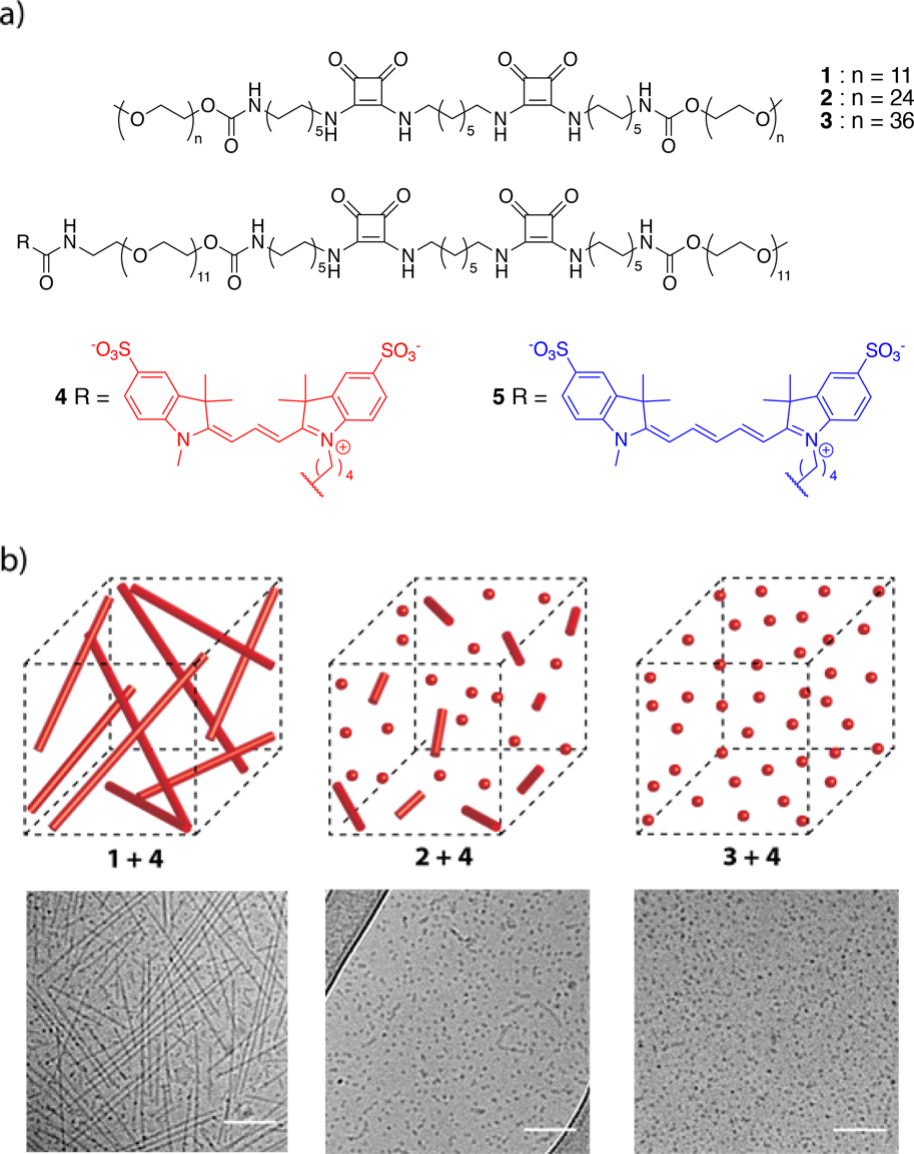
Squaramide-based bolaamphiphiles used to prepare supramolecular polymer nanoparticles for in vivo biodistribution studies. (a) Chemical structure of bolaamphiphile monomers **1**, **2**, and **3** employed in the study to form fibrillar (**1**), mixed rod and spherical (**2**), or spherical (**3**) nanoparticles, respectively, upon self-assembly. The fluorescently labeled squaramide-based monomer with a sulfo-Cy3 dye (**4**) is used for tracking of the various supramolecular polymer nanostructures in vivo, while the sulfo-Cy5 dye (**5**) is used in combination with **4** for fluorescence resonance energy transfer (FRET) experiments. (b) Schematic representation of the morphology of the co-assembled native (**1**, **2**, or **3**) with dye-labeled monomer **4** (2 mol %) into fluorescently labeled squaramide-based supramolecular polymer nanoparticles before injection into a zebrafish embryo model. Representative cryo-TEM images of co-assembled squaramide-based supramolecular polymer nanoparticles from native monomers (2 mM) **1**, **2**, and **3** with **4** in water. Samples were prepared using the native squaramide-based bolaamphiphile monomer co-assembled with 2 mol % **4**, displaying fiberlike structures for **1**, a mixture of spherical and rodlike structures for **2** and spherical aggregates for **3**. The scale bars represent 100 nm.

Supramolecular self-assembly can provide efficient access to a wide range of soft nanostructures, including those that are anisotropic through the association of polymer precursors or designed monomers using noncovalent interactions such as hydrogen bonding, aromatic interactions, and electrostatic and/or hydrophobic effects.^19−21^ Such an approach is particularly attractive for applications in drug delivery because of its facile and modular character, where functional monomers can be combined in a mix-and-match fashion to prepare designed nanocarriers, including those that are anisotropic, against a wide range of therapeutic targets.^22−31^ We previously reported a series of squaramide-based bolaamphiphiles **1**–**3** (Figure 1a) that self-assemble in water to form aggregates with nanoscale dimensions.^32,33^ Squaramides^34,35^ are supramolecular synthons that consist of a cyclobutenedione ring with two NH hydrogen bond donors opposite two carbonyl hydrogen bond acceptors and are attractive for use in materials because of their minimalistic character and ease to introduce synthetically.^36−39^ We found that these self-assembled aggregates can be tuned in shape from fibrillar to spherical when the steric demand of the hydrophilic oligo(ethylene glycol) chains on the monomer was increased with chain length (*n*=11–36). We herein disclose the fluorescent labeling of the squaramide supramolecular polymer nanoparticles by co-assembly of the monomers with a sulfo-Cy-labeled monomer for their in vivo tracking in a zebrafish embryo model (Figure 1a). More specifically, we study their in vivo biodistribution behavior and the capacity to tune their interaction with LSECs through the Stabilin-2 receptor by the choice of monomers and their resultant aggregate structures.

## Experimental Section

### General

All reagents and chemicals were purchased from commercial sources and used without further purification. Sulfo-Cy3 and sulfo-Cy5 dyes were obtained from Lumiprobe, while oligo(ethylene glycols) of various chain lengths (*n* = 11, 24, and 36) were purchased from Broadpharm. Monomers **1**, **2**, and **3** were synthesized as previously reported (Scheme S1).^33^ The synthetic protocol for the sulfo-Cy3 and sulfo-Cy5 squaramide-based bolaamphiphiles **4** and **5**, respectively, can be found in the Supporting Information. Milli-Q water was used for all experiments. ζ-Potential measurements were performed on a Zetasizer Nano ZS (Malvern). UV–vis measurements were carried out on a Cary 300 UV–vis spectrophotometer using a quartz cuvette with a path length of 1 cm. Fluorescence experiments were executed on an Infinite M1000 Pro Tecan plate reader using 96-well plates with a black background.

### Preparation Protocol of Co-Assembled Sulfonated Cyanine-Labeled Squaramide-Based Supramolecular Polymer Nanoparticles

Stock solutions of **1**, **2**, or **3** were prepared at a concentration of 5.8 mM in DMSO, while stock solutions of **4** or **5** were prepared at a concentration of 0.2 mM in the same solvent. The native (**1**, **2**, or **3**) and dye-labeled monomers (**4** or **5**) were mixed in a glass vial at the appropriate molar ratios (1 or 2 mol %). The solvent was lyophilized and the bolaamphiphile mixture was reconstituted in water at the desired concentration. The resulting clear solutions were left to stand for 24 h before any measurement.

### Cryogenic Transmission Electron Microscopy (Cryo-TEM)

Samples in water consisting of **1**, **2**, or **3** (2 mM) co-assembled with reporter molecule **4** (2 mol %) were prepared according to the co-assembly protocol above. The samples were left to stand for 24 h before deposition on a glow-discharged grid. Samples of **1** co-assembled with **4** (2 mol %) with increasing concentration of carp serum were prepared following a similar protocol. Instead of adding fresh water after removal of DMSO by lyophilization, a freshly prepared carp serum solution in water was added in the desired v/v% (ranging from 1 to 25% v/v) to provide a final concentration of 2 mM of the fluorescently labeled supramolecular polymer nanoparticle and left to stand 24 h before deposition on a glow-discharged grid. Cryo-TEM samples were prepared by depositing the sample (3 μL) on a glow-discharged lacey carbon film (300-mesh Cu grids). The excess sample was removed by blotting for 1 s at room temperature with 95% humidity (Whatman no. 4 filter paper), and the resulting films were vitrified at −183 °C using a Leica EMGP. Images of the samples were recorded with a Tecnai F20 FEG (FEI), equipped with a field emission gun at 200 keV using a Gatan UltraScan camera with a defocus between −3 and −10 μm.

### UV–Vis Spectroscopy

UV–vis samples in water consisting of **1**, **2**, or **3** (30 μM) co-assembled with **4** (2 mol %) were prepared according to the co-assembly protocol described above. The samples were left to stand for 24 h before UV–vis measurements.

### Fluorescence Spectroscopy (FRET Measurements)

The dye-labeled supramolecular polymer nanoparticles were prepared according to the co-assembly protocol mixing **1**, **2**, or **3** (*c* = 30 μM) with **4** and/or **5** in different concentrations depending on the experiment. For static measurements, **4** and **5** (300 nM each) were mixed together in an equimolar ratio with the native monomer (**1** or **3**) to obtain a total of 2 mol % of the fluorescently labeled molecules in DMSO before lyophilization. After the reconstituted samples in water were left to stand overnight, they were loaded in a fluorimeter and excited at 550 nm (sulfo-Cy3) and their fluorescence emission was measured from 570 to 800 nm (sulfo-Cy5) at room temperature. Samples were measured in triplicate. For dynamic measurements, **4** or **5** (600 nM) was mixed individually with the native monomer (**1**, **2**, or **3**) to obtain mixtures of 2 mol % of the fluorescently labeled molecule in DMSO before lyophilization. The equilibrated samples of the sulfo-Cy3- and sulfo-Cy5-labeled squaramide-based supramolecular polymer nanoparticles were mixed in a 1:1 ratio (100 μL each) in a 96-well plate by pipetting (2–3×) with the acquisition of fluorescence data immediately after mixing at room temperature using excitation at 550 nm (sulfo-Cy3) and measuring fluorescence emission from 570 to 800 nm (sulfo-Cy5). The FRET ratio is the relative fluorescence intensity of the peaks at 670/570 nm. Experiments were run in triplicate to ensure reproducibility. Raw fluorescence data are provided in Figures S5.2 and S5.3, and a control experiment to quantify sulfo-Cy5 emission at an excitation wavelength of 550 nm is described in Figure S5.1.

### ζ-Potential Measurements

Samples for electrophoretic mobility experiments were prepared from either **1** alone, or **1**, **2,** or **3** (2 mM) co-assembled with **4** (2 mol %) as outlined in the co-assembly protocol above. The samples were then transferred to a reusable ζ-potential dip cell before measurements. The samples were left to stand for 24 h before ζ-potential measurements.

### Zebrafish Husbandry and Injections

Zebrafish (*Danio rerio*) were maintained and handled according to the guidelines from the Zebrafish Model Organism Database (http://zfin.org) and in compliance with the directives of the local animal welfare committee of Leiden University. Transgenic *Tg(kdrl:EGFP)*^*s*843^,^40^*Tg(mpeg:EGFP)*^*gl*22^,^41^ and *stab*2^*ibl*2 ^^5^ zebrafish were used. Fertilization was performed by natural spawning at the beginning of the light period, and eggs were collected and raised at 28.5 °C in egg water (60 μg/mL Instant Ocean sea salts). Pigment cell formation was suppressed by adding 1-phenyl-2-thiourea (PTU) to the egg water in 1-day old zebrafish (24–28 hpf).

Fluorescently labeled squaramide-based supramolecular polymer nanoparticles were injected into 2-day old zebrafish embryos (52–56 hpf) using a modified microangraphy protocol described previously.^9^ Embryos were anesthetized in 0.01% tricaine and embedded in 0.4% agarose containing tricaine before injection. Squaramide-based supramolecular polymer nanoparticles (1 nL) consisting of native monomer **1**, **2**, or **3** (*c* = 2 mM) co-assembled with **4** (2 mol %) were injected with a microneedle into the Duct of Cuvier. Successfully injected embryos were identified (damaged-yolk ball embryo excluded) and imaged using a Leica TCS SPE confocal microscope. Confocal micrographs (Z-stacks) for the whole embryo were generated using a 10× air objective (HCX PL FLUOTAR) and overlapping three images to cover the complete embryo; for the caudal vein, a 40× water-immersion objective (HCX APO L) was used. Images were processed using the Fiji Image J software.

## Results and Discussion

### Synthesis and Co-Assembly of Fluorescently Labeled Supramolecular Polymer Nanoparticles

The general molecular structure of the squaramide-based bolaamphiphile consists of two squaramides located within its hydrophobic core surrounded by two hydrophilic oligo(ethylene glycol) oligomers (OEG) at its opposite ends (**1**, **2**, and **3**, Figure 1). The two squaramide synthons are separated by an alkyl chain of seven methylene units, with an additional chain of ten methylene units at the outer peripheries of the squaramide moieties. In this work, hydrophilic OEGs of increasing chain length (**1**: *n* = 11, **2**: *n* = 24, and **3**: *n* = 36) were used to flank the hydrophobic core to drive the formation of supramolecular polymer nanoparticles of distinct shapes and sizes while providing a means to maintain the same surface chemistry.

To be able to tag and track the various supramolecular polymer nanoparticles for their visualization in the transparent zebrafish, an asymmetrically labeled squaramide-based bolaamphiphile, similar in molecular structure to **1**, with either a fluorescent sulfo-Cy3 (**4**) or sulfo-Cy5 (**5**) dye was synthesized. These fluorescently labeled squaramide-based bolaamphiphiles are anticipated to co-assemble with the monomers used to prepare the squaramide-based nanoparticles owing to their equally sized hydrophobic domains, despite their distinct hydrophilic domains. Therefore, a single reporter monomer could be used for the labeling of the various supramolecular polymer nanoparticles under study.

Sulfonated variants of the cyanine dyes were introduced at one end of the bolaamphiphile because of their increased solubility, to reduce the potential for disruption or aggregation of the supramolecular polymer. The synthesis of the asymmetrically labeled fluorescent squaramide-based bolaamphiphile was performed in a convergent manner, on one hand starting from *O*-(2-azidoethyl)undecaethylene glycol and on the other from *O*-methyl-undecaethylene glycol, building inward to prepare the heterobifunctional squaramide-based bolaamphiphile (see the Supporting Information). The sulfonated dyes were subsequently coupled through Boc deprotection of the terminal amine of the bolaamphiphiles and reacted with the corresponding sulfo-Cy3 or sulfo-Cy5 NHS ester under basic conditions to yield **4** or **5**.

The fluorescently labeled supramolecular polymer nanoparticles were prepared first through co-assembly of the native monomer (**1**, **2**, or **3**) and the sulfo-Cy3 or sulfo-Cy5 dye-labeled squaramide-based bolaamphiphile (**4** or **5**, 2 mol %) in DMSO. The samples were lyophilized, reconstituted in water at room temperature and left to stand for 24 h before subsequent measurements. This mix-and-match preparation protocol involving monomer co-assembly can be used to efficiently prepare a library of supramolecular polymer nanoparticles with various physicochemical properties for applications in drug delivery.

In cryo-TEM, the co-assembly of **1** and **4** largely resulted in highly disperse fibrillar objects of length 282 ± 85 nm, with a diameter of 6 ± 1 nm (Figures 1b and S3.1), and a small population of spherical aggregates, with a diameter of 8.2 ± 1 nm (Figure S3.2). The co-assembly of monomers **2** and **4** displayed a mixture of spherical and rodlike aggregate structures, similar to the self-assembly of **2** on its own with a diameter of 6 ± 1 nm and comparable to the co-assembly of monomers **1** and **4** (Figures 1b, S3.3, and S3.4). Conversely, supramolecular polymer nanoparticles of **3** and **4** displayed spherical objects in solution with a diameter of 9 ± 2 nm (Figures 1b and S3.4). The measured dimensions of the supramolecular polymer nanoparticles with the dye-labeled monomers were in agreement with cryo-TEM measurements of the self-assembled native monomers **1**, **2**, and **3**, indicating that they were unaffected by their incorporation.

**Figure 2:**
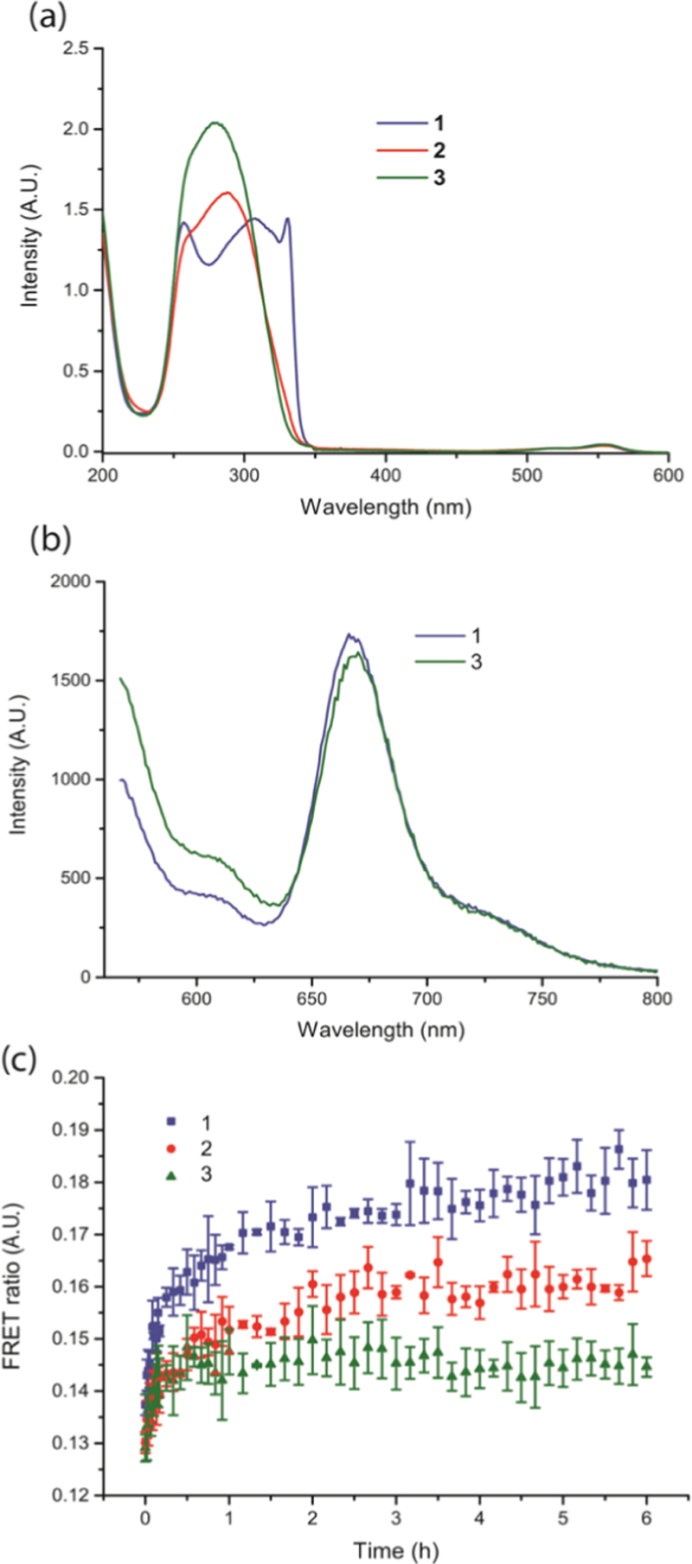
Spectroscopic studies of the self-assembled squaramide-based supramolecular polymer nanoparticles (30 μM). (a) UV–vis spectra of **1** (blue), **2** (red), and **3** (green) co-assembled with **4** (2 mol %). (b) Static measurements of **1** and **3** with an equimolar amount of **4** and **5** (2 mol % total of the dye-labeled monomers); FRET signal observed at 670 nm. (c) Dynamic measurements of **1**, **2**, and **3** co-assembled with **4** or **5** (2 mol % of each dye-labeled monomer) independently and then mixed together in an equimolar ratio. FRET ratios are compared over time (6 h) for **1** (blue squares), **2** (red circles), and **3** (green triangles).

UV–vis spectroscopy measurements further supported the lack of disruption of the supramolecular polymer architecture at the molecular level after co-assembly of **4** with the various native monomers at concentrations above their critical aggregation concentration (Figures 2a and S4.2). Co-assembly of **1** and **4** showed UV–vis spectra comparable to those of native fibrillar self-assemblies of **1** with bands at 255 and 329 nm, corresponding to the HOMO–(LUMO + 1) and HOMO–LUMO transitions of the squaramide,^32^ respectively (Figures 1a, S5.2, and S5.3). Similar spectral traces were recorded for **2** with **4**, and **3** with **4** at the same molar ratio, with a lower degree of blue- and red-shifting of the HOMO–(LUMO + 1) and HOMO–LUMO bands when compared against **1** with **4**, especially for **3**. Concentration-dependent UV–vis spectra of **3** from 0.3 to 67 μM showed negligible changes in the absorption profile even when concentrations of the monomer above the critical aggregation concentration were measured (Figure S5.1). Moreover, spectral data for **2** and **3** with **4** are on a par with those obtained for the unfunctionalized monomers that showed a decrease or absence of the band at 329 nm and a shoulder at 255 nm.^33^ Collectively, these results suggest that the supramolecular polymer nanoparticles largely retain their aggregation even after the co-assembly of the fluorescent monomer.

Fluorescence resonance energy transfer (FRET) measurements were used to study incorporation of dye-labeled monomers into the supramolecular polymer nanoparticles (static measurements) and the rate of monomer exchange between nanoparticles (dynamic measurements) (Figures 2, S6.2, and S6.3). Static measurements were performed to evaluate the colocalization of monomers labeled with a sulfo-Cy3 dye (molecule **4**, FRET donor) and those with a sulfo-Cy5 dye (molecule **5**, FRET acceptor) at 1 mol % each with native monomer **1** or **3**. The co-assembly of monomer **1** or **3** with **4** and **5** resulted in an intermediate degree of FRET efficiency in comparison to other supramolecular polymers^42,43^ by examining the acceptor emission at λ_em_ ≈ 670 nm (Figure 2b) against the fluorescence intensity of the donor emission λ_em_ ≈ 570 nm relative to a Cy5 control (Figures S6.3 and S6.1). Moreover, a slightly higher degree of FRET efficiency was found for fibers of **1** in comparison to spherical aggregates of **3** and is consistent with their morphological differences. Overall, these results indicate that incorporation of both dye-labeled molecules into the supramolecular polymer nanoparticles occurs despite the distinct size of the hydrophilic domains presented by **1** or **3**.

Dynamic experiments were subsequently performed to understand the rate of monomer exchange between the supramolecular polymer nanoparticles. In these experiments, fluorescently labeled supramolecular polymer nanoparticles were first prepared by the co-assembly of native monomer **1**, **2**, or **3** with 2 mol % of **4** or **5**. The distinctly labeled fluorescent nanoparticles with sulfo-Cy3 or sulfo-Cy5 were mixed and monomer colocalization within the fibers was measured by the evolution in FRET intensity over time. Monomer exchange between assemblies of **1**, **2**, or **3** with **4** (sulfo-Cy3) and assemblies (**1**, **2**, or **3**) labeled with reporter molecule **5** (sulfo-Cy5) was observed with different kinetic profiles. Assemblies of **3** appeared to reach a plateau after 1 h, suggesting that the sample attained an equilibrium state in its exchange, whereas for **1**, a plateau was still not reached after 6 h. Qualitatively, the curves show a faster exchange of monomers in the case of the spherical aggregates (green line) composed of **3** compared to the fibrillar objects of **1** (blue line) (Figure 2c). In all cases, the significantly lower FRET ratios observed in dynamic compared to the static measurements suggest that slow dynamics or a partial exchange of monomers occurs within the fibers, but FRET experiments show the effective labeling of the supramolecular polymer nanoparticles by the sulfo-Cy monomers.

Because the sulfonated cyanine dyes used to fluorescently tag and track the supramolecular polymer nanoparticles bear a negative charge, zeta (ζ) potential measurements were performed to estimate the surface charge of the aggregates in water. The co-assembly of native monomer **1**, **2**, or **3** with **4** (2 mol %) resulted in near-neutral ζ-potential values of −4.9 ± 5.2, −12.3 ± 4.7, and −11.1 ± 4.5 mV, respectively. As controls, native monomer **1** (*c* = 2 mM) on its own showed similar ζ-potential values of −7.32 ± 5.5 mV and, thus, indicated that its co-assembly with monomer **4** was comparable in surface charge with the native self-assembled constructs. Moreover, the negative ζ-potential value of −32.1 ± 7.1 mV recorded for **4** on its own, which has a fibrillar morphology (*c* = 40 μM) in cryo-TEM measurements, was not recorded for the mixtures and suggested that monomer co-assembly occurred in the final aggregates. Hence, all investigated supramolecular polymer nanoparticles co-assembled with an anionic dye-labeled monomer were near-neutral in surface charge in comparison to **4** on its own that was negative.

Cumulatively, we demonstrate the co-assembly between a single sulfonated cyanine (**4** or **5**)-labeled fluorescent bolaamphiphile and various native squaramide-based bolaamphiphiles of increasing OEG hydrophilic side chain lengths (**1**, *n* = 11; **2**, *n* = 24; and **3**, *n* = 36) to prepare fluorescently tagged supramolecular polymer nanoparticles ranging from fibrillar to spherical with increasing size of the hydrophilic domain. This mix-and-match strategy considerably reduces the synthetic effort to obtain libraries of functional assemblies by using a single cargo-loaded molecule that co-assembles with a variety of monomers.

### In Vivo Evaluation of Squaramide-Based Supramolecular Polymer Nanoparticles

Before the evaluation of the in vivo biodistribution behavior of the supramolecular polymer nanoparticles in a zebrafish embryo, their stability was first assessed in biological media by monitoring their morphology in the presence of increasing concentrations of carp serum (CS). Carp serum was used as the closest practical approximation to zebrafish serum. Diluted concentrations of CS, ranging from 1 to 25% v/v, were probed to enable differentiation of the supramolecular polymer nanoparticles from the high background imposed by the serum components in cryo-TEM (Figure S7.1) measurements. Even in the presence of increasing CS concentrations, the supramolecular polymer nanoparticles consisting of **1** and **4** retained their respective morphologies and sizes relative to self-assemblies of the native monomer **1** (around several hundreds of nanometers in fiber length and a width between 5 and 6 nm), indicating that the serum components did not affect the co-assemblies (Figures S7.2–S7.5). This result is likely due to the high density of ethylene glycol oligomers, known to increase the circulation time by reducing the interaction of nanoparticles with plasma proteins or their opsonization,^44−46^ on the periphery of the supramolecular polymer nanoparticles. However, samples of **2** or **3** with carp serum solutions of varied concentrations could not be discriminated from the serum background in the electron micrographs that is high in protein content with nanostructured objects of the same size (25–300 nm)^47^ and contrast to the squaramide-based aggregates.

Zebrafish embryos between 52 and 56 hpf (hours post-fertilization) were used to establish the squaramide-based nanoparticles as potential drug carriers in vivo. At this stage, blood circulation is robust and most organs are established. Subsequently, monomer solutions of **1**, **2**, and **3** were injected into the zebrafish embryos at a concentration of 2 mM to retain their aggregation with respect to their estimated 30-fold dilution in the serum volume.^48,49^ The biodistribution of the supramolecular polymer nanoparticle solutions in the various zebrafish embryos was imaged 1 h after injection based on our earlier work that demonstrated rapid sequestration of anionic nanoparticles (≪1 h) in the caudal vein after injection.^5^

**Figure 3:**
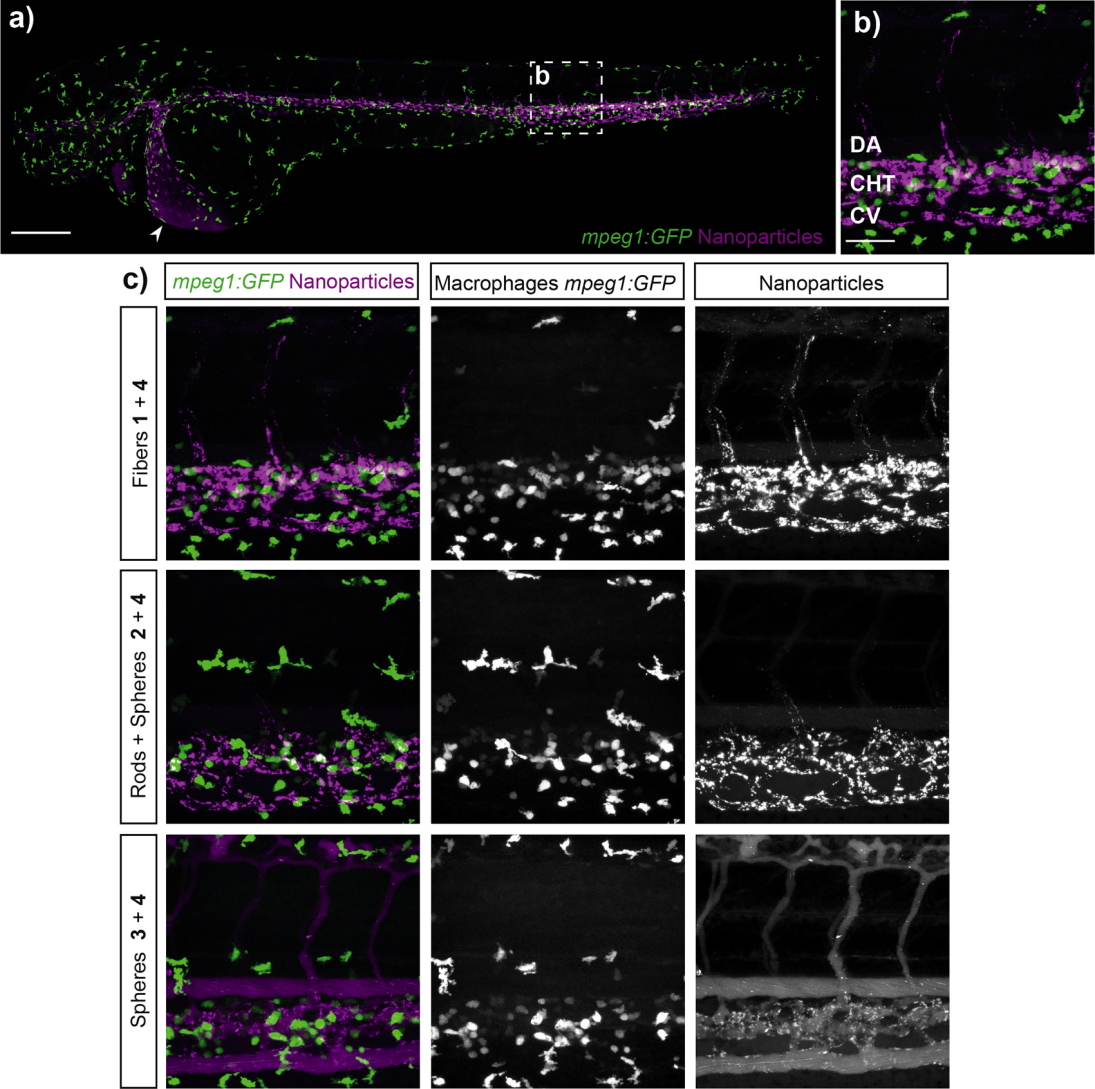
Squaramide-based supramolecular polymer nanoparticles avoid macrophage uptake after 1 h of intravenous injection. (a) Whole-embryo view (10× magnified) after 1 h of injection in the Duct of Cuvier of **1** (2 mM) co-assembled with **4** (2 mol %) in an transgenic zebrafish (*mpeg*1*:EGFP*^*g*l22^) expressing *EGFP* in macrophages (green) at 56 hpf. The arrow represents the site of injection (Duct of Cuvier). Scale bar represents 200 nm. (b) Higher magnification (40×) of the boxed region in (a) showing the dorsal aorta (DA), caudal hematopoietic tissue (CHT), and the caudal vein (CV). Scale bar represents 50 nm. (c) Biodistribution of fluorescently labeled squaramide-based supramolecular polymer nanoparticles 1 h post-injection. Left panel represents an overlay of macrophage and fluorescently labeled squaramides (magenta) of **1** (top), **2** (middle), and **3** (bottom) (2 mM) with **4** (2 mol %).

Macrophage uptake was first considered since it is well established that this cell type plays an important role in processing nanoparticles, often hindering drug delivery in vivo through particle clearance.^5,50,51^ Thus, transgenic zebrafish *Tg(mpeg*1*:EGFP)*^*gl*22^ embryos in which *EGFP* is expressed in macrophages were used, where colocalization of the fluorescent squaramide-based supramolecular polymer nanoparticles would indicate uptake of or binding to macrophages after intravenous injection. Remarkably, no significant colocalization of the supramolecular polymer nanoparticles of any shape and macrophages in the injected zebrafish (after 1 h) was observed (Figures 3 and S8.1). This result is consistent with the earlier measurements of the supramolecular polymer nanoparticles in carp serum and suggests that the decreased protein absorption aids in the evasion of macrophages by the squaramide-based supramolecular polymer nanoparticles.

**Figure 4:**
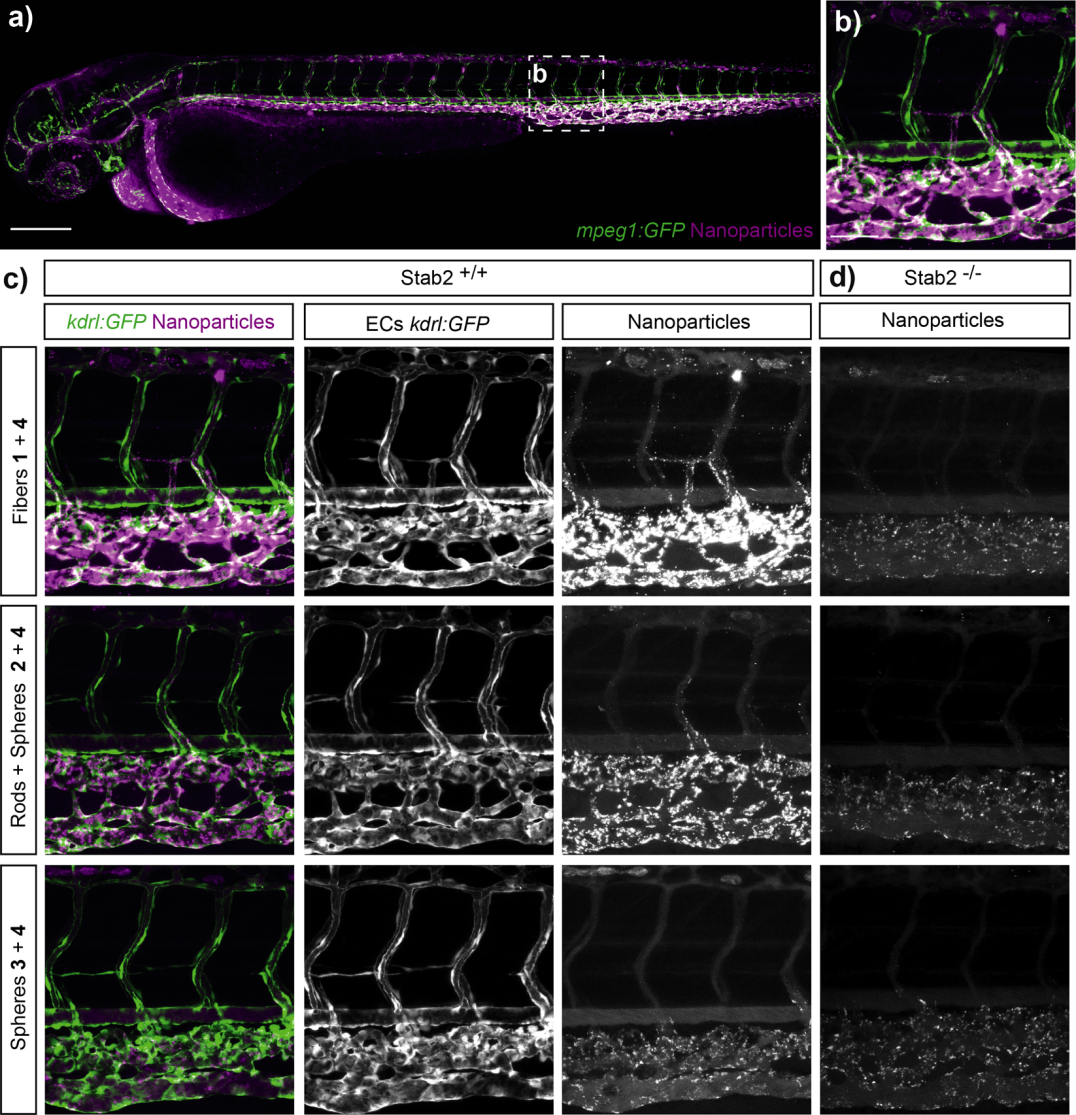
Biodistribution of the fluorescently labeled squaramide-based supramolecular polymer nanoparticles in the *Tg*(*kdrl:GFP*) zebrafish expressing *GFP* in endothelial cells. (a) Whole-embryo view 1 h post-injection in the Duct of Cuvier of **1** (2 mM) with **4** (2 mol %) in embryonic zebrafish at 56 hpf. (b) Higher magnification (40×) of the boxed region in (a) (caudal vein region) showing the dorsal aorta (DA), caudal hematopoietic tissue (CHT), and the caudal vein (CV). Scale bar represents 200 nm. (c) Biodistribution of **1**, **2**, and **3** fluorescently labeled 1 h post-injection. In the left panel, schematic representation of **1** (top), **2** (middle), and 3 (bottom) (2 mM) with **4** (2 mol %). (d) Biodistribution of **1** (top), **2** (middle), and **3** (bottom) (2 mM) with **4** (2 mol %) in the zebrafish mutant *stab*2^*ibl*2^ (stab2^–/–^).

Still, most of the anisotropic supramolecular assemblies were rapidly removed from circulation (Figure 4), associating largely with the endothelial cells in the CV. Most strikingly, the elongated fibrillar objects composed of the co-assembly of **1** and **4** showed limited free circulation, with a preference for accumulation in a subset of venous endothelial cells (Figures 4a,b and S8.2). Recently, we have shown that these cells in the zebrafish embryo are functionally homologous to the LSECs in mammals.^5^ In both zebrafish and mammals, these cells are specialized in endocytosis of macromolecular and nanoparticulate waste from circulation.^52^ Within these cells, the transmembrane scavenger receptor, Stabilin-2, plays a major role in mediating uptake of nanoparticles with anionic character.^5,53,54^

When we analyzed the nanoparticle distribution of the co-assembled supramolecular polymer nanoparticles composed of **1** and **4** in mutant embryos lacking functionality of the Stabilin-2 receptor (*stab*2^*ibl*2^),^5^ their uptake by scavenger endothelial cells was largely abrogated, displaying an increased circulation lifetime. Similar results were observed for supramolecular polymer nanoparticles consisting of **2** and **4**, although their structure already displayed reduced uptake and increased circulation time relative to a co-assembly of **1** and **4** in wild-type embryos. Interestingly, for the co-assembly of **3** and **4**, only a slight accumulation in SECs was observed in the *stab*2^*ibl*2^ mutant embryos and its uptake, therefore, appeared less dependent on Stabilin-2 function (Figure 4b). Thus, Stabilin-2-mediated nanoparticle uptake by SECs is not only influenced by surface charge^5^ but also by nanoparticle shape and/or size, with a preference for the larger anisotropic particles. Shape-dependent circulation and distribution were reported for several types of nanoparticles in mammalian models^1,55^ but were mainly ascribed to differences in their hydrodynamic properties lacking a view into their sequestration by this receptor. This study shows, for the first time, the potential to tune the distribution behavior of supramolecular polymer nanoparticles of distinct shape and size and their uptake by the Stabilin-2 receptor within the zebrafish embryo model. Moreover, these results suggest that new chemical designs or methods of application of supramolecular polymer nanoparticles in vivo are necessary, if liver targeting is not desired, to benefit from the advantages earlier demonstrated for fibrillar nanoparticles, such as longer circulation times and decreased toxicity.^55,56^

## Conclusions

A mix-and-match co-assembly approach starting from bolaamphiphilic monomers consisting of identical chemical units but with distinct hydrophilic domain sizes, and a fluorescently labeled bolaamphiphile, was used to prepare fluorescent supramolecular polymer nanoparticles of distinct shape and size while maintaining their surface chemistry. These supramolecular polymer nanoparticles retained their shape and size after co-assembly with the fluorescent monomer, showed dynamic monomer exchange in FRET experiments, had near-neutral surface charge, and remained self-assembled in the presence of complex biological media, such as carp serum. Tunable in vivo biodistribution behavior of these squaramide-based supramolecular polymer nanoparticles was readily visualized in optically transparent zebrafish embryos. Fibrillar morphologies displayed a low circulation time with rapid association with venous endothelial cells, whereas spherical nanoparticles demonstrated significantly greater mobility with longer circulation times and improved distribution over the zebrafish.

These observations were rationalized by the clearance of the samples containing fibrillar particles by the SECs through an interaction with Stabilin-2; however, the low fluorescence observed in the CV for the *stab*2 knockouts with the spherical particles points out the need to further investigate this process. Moreover, it was found that these supramolecular polymer nanoparticles also evaded macrophages. These results demonstrate that the nanocarrier shape and size, even at very high aspect ratios, play an important role in nanoparticle clearance by SECs through the Stabilin-2 receptor. In addition, we highlight the use of the zebrafish as a convenient model to mechanistically study the biodistribution of fluorescent supramolecular polymer nanoparticles with the examination of receptor-mediated interactions, as well as a fast and cost-effective in vivo prescreening tool for nanomedicines so as to bridge the current gap between in vitro and in vivo studies involving rodents.^6^ It is anticipated that by understanding the in vivo routing of such fibrillar nanoparticles at the cellular level and establishing their structure–activity relationships, new chemical and/or biological strategies can be developed to improve payload delivery to a particular target.

## Supporting Information Available

Synthetic details of all of the reported compounds, spectroscopic data, and cryo-TEM and zebrafish images (PDF)
