# Supplementary material for: Stab2-Mediated Clearance of Supramolecular Polymer Nanoparticles in Zebrafish Embryos
Source: Biomacromolecules. 2020 Feb 21;21(3):1060–8. doi: 10.1021/acs.biomac.9b01318 (PMC9665412; doi:10.1021/acs.biomac.9b01318)
Supplement: Supplementary file 1 — bm9b01318_si_001.pdf [file bm9b01318_si_001.pdf]

Supporting Information

Stab-2 mediated clearance of supramolecular polymer nanoparticles in zebrafish embryos

Victorio Saez Talens[†]*, Gabriela Arias-Alpizar*[†]*, D. M. M. Makurat, Joyal Davis, Jeroen Bussmann, Alexander Kros* and Roxanne E. Kieltyka**

Supramolecular and Biomaterials Chemistry, Leiden Institute of Chemistry, Leiden University, P.O. Box 9502, 2300 RA Leiden, The Netherlands

†Contributed Equally

1. General 2

2. Synthetic route 3

3. Cryo-TEM 7

4. Critical aggregation concentration determination 11

5. UV-Vis spectra 13

6. Fluorescence spectra 14

7. Cryo-TEM of 1 with carp serum 16

8. Zebrafish husbandry, injections and whole embryo imaging 18

9. References 20

1. General

Deuterated dimethylsulfoxide and chloroform were purchased from Euriso-top. The reaction intermediates obtained in the synthesis of the squaramide-based bolaamphiphiles were purified using a Grace Reveleris X1 flash chromatography system equipped with a C18 column, while the final sulfonated cyanine-labeled squaramide-based bolaamphiphiles (**4** and **5**) were purified by RP-HPLC with a Vydac C18 reversed phase column. ^{1}H NMR and ^{13}C NMR spectra were acquired on a Bruker AV-III-600 MHz at 298K. LC-MS analysis was performed with a TSQ Quantum Access MAX system equipped with a Gemini 3 µm C18 110 Å 50 × 4.60 mm column (UV detection at 214 nm and 254 nm). Light scattering data was collected on Malvern Zetasizer Nano ZS ZEN3500. The laser wavelength was 633 nm and scattering angle 173 °.

2. Synthetic route

The synthetic procedure for the sulfonated cyanine-labeled squaramide-based bolaamphiphiles is shown below. Squaramide-based bolaamphiphiles **1**, **2** and **3**, and intermediates **6** and **7** were reported elsewhere.[1, 2]

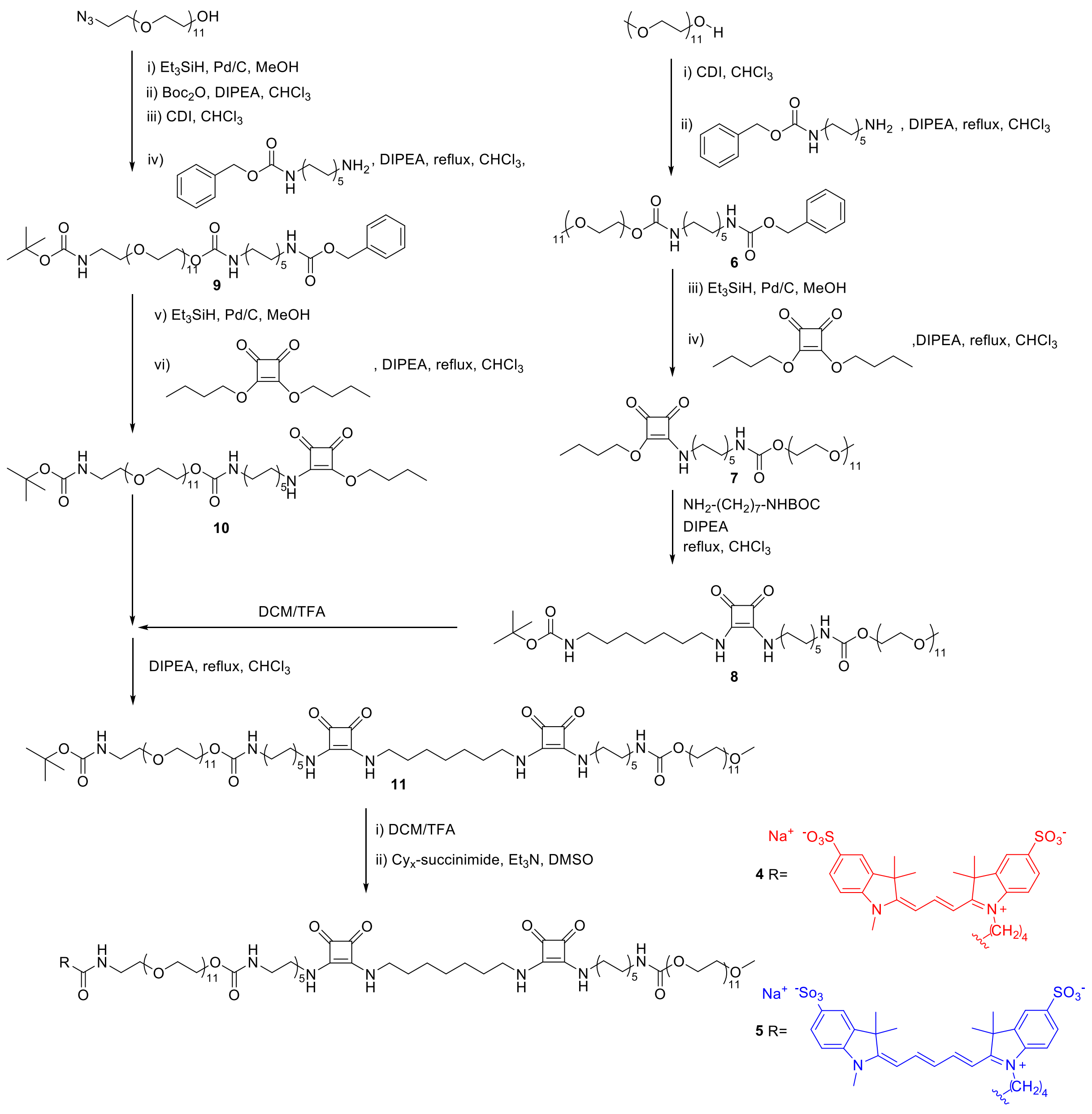

Scheme S1. Synthetic route for sulfonated cyanine-labeled monomers **4** and **5**.

Synthesis of 8

N-Boc-1,7-diaminoheptane (41.4 mg, 0.18 mmol) (synthesis reported elsewhere[3]) was added to a stirred mixture of **7** (106.0 mg, 0.12 mmol) and DIPEA (105.0 µL, 0.60mmol) in $CHCl_3$ (10 mL) refluxed overnight. The resulting compound was purified by reversed phase C18 silica column using a gradient

of 10-90% CH_3CN/H_2O over 45 minutes. The product was concentrated by evaporation and lyophilized to obtain a white solid.

Yield: 70.1 mg, 56.0% ^{1}H-NMR (δ_H[ppm], $CDCl_3$, 600 MHz): 7.46 (br s, 1H), 5.09 (br s, 1H), 4.83 (br s, 1H), 4.20-4.19 (m, 2H), 3.84-3.65 (m, 44H), 3.56-3.52 (m, 2H), 3.38 (s, 3H), 3.15-3.06 (m, 4H), 1.64-1.17 (m, 35H).^{13}C-NMR (δ_C[ppm], $CDCl_3$, 150 MHz): 183.43, 183.40, 169.02, 168.98, 157.58, 157.54, 80.10, 72.90, 72.82, 71.56, 71.53, 71.52, 71.49, 71.27, 70.99, 70.65, 64.81, 45.79, 45.60, 45.53, 42.04, 41.47, 32.19, 31.99, 30.94, 30.53, 30.46, 30.44, 30.31, 30.25, 30.23, 29.83, 29.47, 27.71, 27.58, 27.47, 27.37.LC-MS: t =6.67 min, *m/z*: 922.80 $[M+H-Boc]^+$.

Synthesis of 9

O-(2-Azidoethyl)undecaethylene glycol (0.6 g, 1.05 mmol) was dissolved in 5 mL MeOH and Pd/C (55.0 mg, 0.52 mmol) was added followed by briefly degassing the reaction mixture with argon. Triethylsilane (1.7 mL, 10.50 mmol) was added dropwise to the reaction mixture resulting in an effervescent solution, and was left stirring at room temperature for two hours. After deprotection, the intermediate was confirmed by LC-MS (t = 3.65 min, *m/z*: 546.13 $[M+H]^+$). Pd/C was removed by filtration over Celite, the reaction mixture was concentrated by rotary evaporation and dried with gentle stream of compressed air. Subsequently, O-(2-aminoethyl)undecaethylene glycol was protected using Boc-anhydride (0.3 g, 1.37 mmol) and DIPEA (0.55 mL, 3.15 mmol) in $CHCl_3$ (10 mL) upon stirring for 2 hours. The intermediate formation was verified by LC-MS (t = 4.73 min, *m/z*: 545.78 $[M+H-Boc]^+$). Next, 1,1'-carbonyldiimidazole (0.29 g, 1.79 mmol) was added to the reaction mixture with stirring for an additional 2-3 hours to activate the hydroxyl group of the oligo(ethylene glycol) and verified by LC-MS (t = 7.57 min, *m/z*: 639.17 $[M+H-Boc]^+$). N-Cbz-1,10-decanediamine (0.42 g, 1.37 mmol) and DIPEA (0.37 mL, 2.10mmol) was added to the reaction mixture and refluxed overnight. The reaction mixture was purified by reversed phase column chromatography using a 10-90% CH_3CN/H_2O gradient over 40 minutes. The final product was concentrated by evaporation ofCH_3CN followed by lyophilization of waterto obtain compound **9** as a white solid.

Yield: 0.52 g, 50.6% ^{1}H-NMR (δ_H[ppm], $CDCl_3$, 600 MHz): 7.28-7.22 (m, 5H), 5.16 (br s, 1H), 5.07-5.05 (br s, 1H), 5.01 (s, 2H), 4.12 (t, 2H), 3.68-3.54 (m, 42H), 3.46-3.44 (t, 2H), 3.23-3.22 (m, 2H), 3.11-3.04 (m, 4H), 1.41-1.37 (m, 13H), 1.26-1.20 (m, 12H). ^{13}C-NMR (δ_C[ppm], $CDCl_3$, 150 MHz): 156.53, 156.09, 136.86, 128.50, 128.09, 128.04, 79.45, 72.72, 70.60, 70.55, 70.27, 69.70, 66.47, 63.78, 41.14, 41.06, 40.43, 29.97, 29.44, 29.25, 28.51, 26.75. LC-MS: t = 8.26 min, *m/z*: 878.33 $[M+H-Boc]^+$.

Synthesis of 10

Compound **9** (0.52 g, 0.53 mmol) was dissolved in MeOH (5 mL) and Pd/C (28.0 mg, 0.27 mmol) was added followed by briefly degassing the reaction mixture with argon. Triethylsilane (0.85 mL, 5.32 mmol) was added dropwise to the reaction mixture resulting in an effervescent solution, and was left stirring at room temperature for two hours. After Cbz-deprotection, the intermediate was confirmed by TLC-MS (*m/z*: 746.13 $[M+H\text{-}Boc]^+$), Pd/C was removed by filtration over Celite, concentrated by rotary evaporation and dried with gentle stream of compressed air. The crude product was redissolved in $CHCl_3$ (10 mL) and 3,4-dibutoxy-3-cyclobutene-1,2-dione (149.0 µL, 0.69 mmol) and DIPEA (185.0 µL, 1.06 mmol) were added. The reaction mixture was refluxed overnight and subsequently, purified by C18 silica column using a gradient of 10-90% CH_3CN/H_2O over 40 minutes. The final product was concentrated by evaporation of CH_3CN followed by lyophilization of water to obtain a compound **10** as a white solid.

Yield: 0.42 g, 61.1% ^{1}H-NMR (δ_H[ppm], $CDCl_3$, 600 MHz): 5.18 (br s, 1H), 5.05 (br s, 1H), 4.75-4.73 (t, 2H), 4.21-4.20 (t, 2H), 3.76-3.61 (m, 42H), 3.56-3.53 (m, 2H), 3.42-3.37 (m, 2H), 3.31 (br s, 2H), 3.16-3.13 (m, 2H), 1.80-1.77 (m, 2H), 1.62-1.59 (m, 4H),1.48-1.44 (m, 15H), 1.31-1.28 (m, 12H),0.99-0.96 (t, 3H).^{13}C-NMR (δ_C[ppm], $CDCl_3$, 150 MHz): 190.45, 183.89, 178.41, 173.44, 157.47, 157.10, 80.17, 74.40, 72.88, 72.81, 72.64, 72.27, 71.48, 71.37, 71.35, 71.32, 71.25, 71.18, 71.08, 70.98, 70.83, 70.69, 67.91, 64.76, 64.66, 45.88, 45.42, 42.00, 41.34, 41.04, 40.90, 33.03, 32.06, 31.83, 31.63, 30.88, 30.33, 30.16, 30.05, 29.45, 27.66, 27.32, 19.67, 14.70. LC-MS: t = 7.38 min, *m/z*: 995.30 $[M+H\text{-}Boc]^+$.

Synthesis of 11

Trifluoroacetic acid (2 mL) was added to **8** (31.8 mg, 0.031 mmol) to facilitate deprotection of the Boc-protecting group, and verified by LC-MS (t = 6.67min, *m/z*: 922.80 $[M+H\text{-}Boc]^+$) after 20 minutes. Afterwards, the solvent was evaporated by using a gentle stream of compressed air and the compound was redissolved in $CHCl_3$ (5 mL) with DIPEA (0.5 mL), and **10** (40.1 mg, 0.041 mmol) was added prior to refluxing the reaction mixture overnight. The compound was purified by C18 column chromatography using a gradient of 10-90% CH_3CN/H_2O over 25 minutes. The final product was concentrated by evaporation of CH_3CN followed by lyophilization of water to obtain a compound **11** as a white solid.

Yield: 20.4 mg, 35.6% ^{1}H-NMR (δ_H[ppm], $CDCl_3$, 600 MHz): 7.76 (br s, 1H), 7.52 (br s, 1H), 5.04 (br s, 1H), 4.21-4.20 (m, 4H), 3.69-3.54 (m, 94H), 3.39 (s, 3H), 3.32 (br s, 1H), 3.15 (m, 4H), 1.67-1.64 (m, 8H), 1.48-1.27 (m, 45H).^{13}C-NMR (δ_C[ppm], $CDCl_3$, 150 MHz): 183.62, 182.51, 170.00, 168.11, 157.54, 80.19, 72.92, 71.57, 71.54, 71.53, 71.49, 71.25, 70.68, 64.81, 60.04, 45.80, 44.21, 42.08, 41.39, 32.17, 30.98, 30.47, 30.27, 30.25, 29.46, 27.76, 27.43, 25.72. LC-MS: t = 6.73 min, 1745.55 *m/z*: $[M+H\text{-}Boc]^+$.

Synthesis of 4

Compound **11** (4.5 mg, 0.0024 mmol) was deprotected using trifluoroacetic acid (1 mL) for 20 minutes. Deprotection of the Boc-protecting group was confirmed by LC-MS (t= 5.77 min, m/z: 1745.27 $[M+H]^+$) and the solvents were evaporated by a gentle stream of compressed air before being redissolved in DMSO (1.5 mL). Triethylamine (250 µL) was added to the stirred solution along with Sulfo-Cy3 NHS ester (2.12 mg, 0.0036 mmol). MilliQ water (3.5 mL) was added to the reaction mixture and dialyzed against water using a dialysis bag with a molecular weight cut off (MWCO) of 1000 Da. The compound

was purified by reversed phase HPLC using a C18 column and a gradient of 10-90% CH_3CN/H_2O over 25 minutes. The product fractions were collected and lyophilized to yield compound **4** as a sticky pink material.

Yield: 2.36 mg, 45.01%. LC-MS: t = 5.83 min, *m/z*: 2345.1 $[M+H]^+$.

Synthesis of 5

Compound **11** (4.5 mg, 0.0024 mmol) was deprotected using trifluoroacetic acid (1 mL) for 20 minutes. Deprotection of the Boc-group was confirmed by LC-MS (t= 5.77 min, m/z: 1745.27 $[M+H]^+$) and the solvents were evaporated by a gentle stream of compressed air before being redissolved in DMSO (1.5 mL). Triethylamine (250 µL) was added to the stirred solution along with Sulfo-Cy5 NHS ester (2.2 mg, 0.0036 mmol). MilliQ-water was added to the reaction mixture and dialyzed against water using a dialysis bag with a molecular weight cut off (MWCO) of 1000 Da. The compound was purified by reversed phase HPLC using a C18 column and a gradient of 10-90% CH_3CN/H_2O over 25 minutes. The product fractions were collected and lyophilized to yield compound **4** as a sticky dark blue material.

Yield: 2.13 mg, 40.14%. LC-MS: t = 5.87 min, *m/z*: 2372.03 $[M+H]^+$.

3. Cryo-TEM

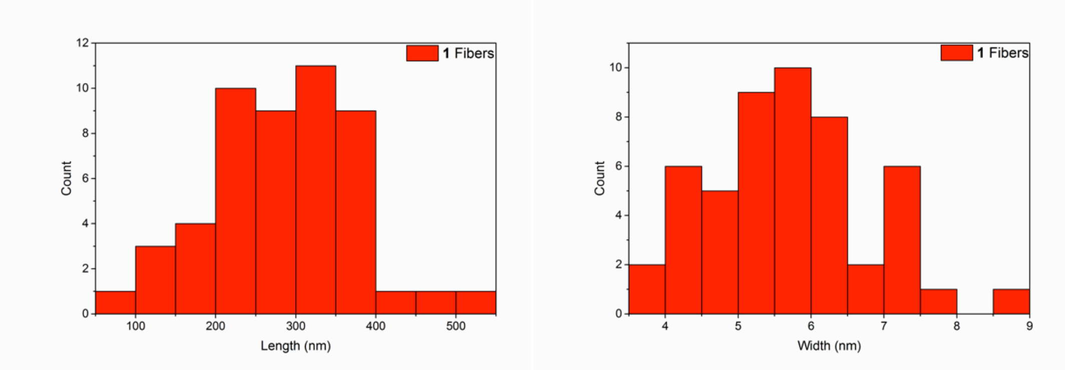

Figure S3.1 Histograms of length (282 ± 84 nm) and width (6 ± 1 nm) distributions measured for fibrillar aggregates of **1** labeled with **4** (n= 50) measured from Cryo-TEM images.

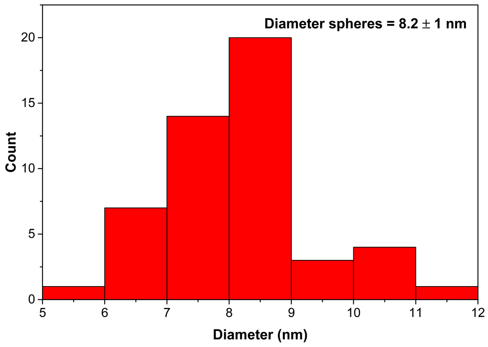

Figure S3.2 Histograms of diameter (8.2 ± 1 nm) distributions measured for spherical aggregates of **1** labeled with **4** (n = 50) measured from Cryo-TEM images.

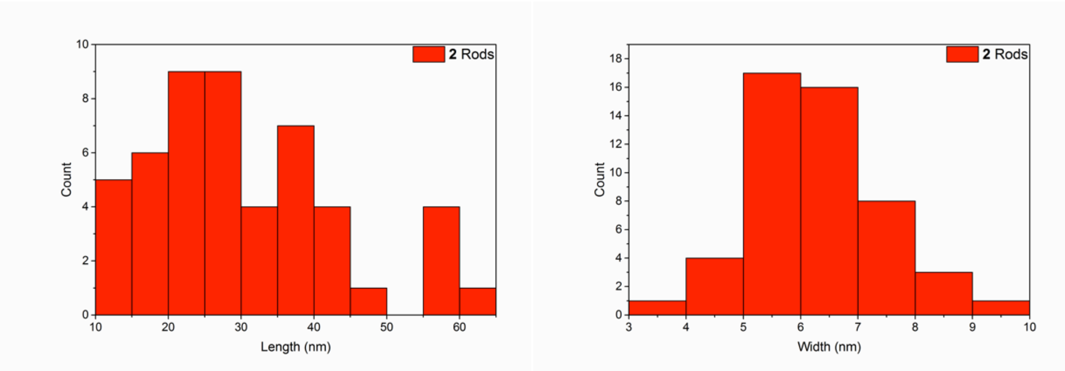

Figure S3.3 Histograms of length (31 ± 13 nm) and width (6 ± 1 nm) distributions measured for **2** labeled with **4** (n= 50) measured from Cryo-TEM images.

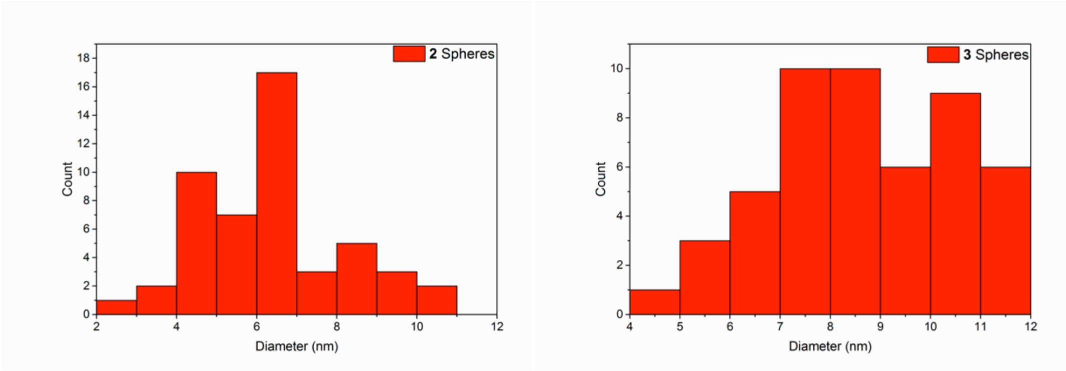

Figure S3.4 Histograms of diameter distributions measured for spherical aggregates of **2** (6 ± 2 nm)(*left*) and **3** (9 ± 2 nm) (*righ*t) labeled with **4** (n= 50) measured from Cryo-TEM images.

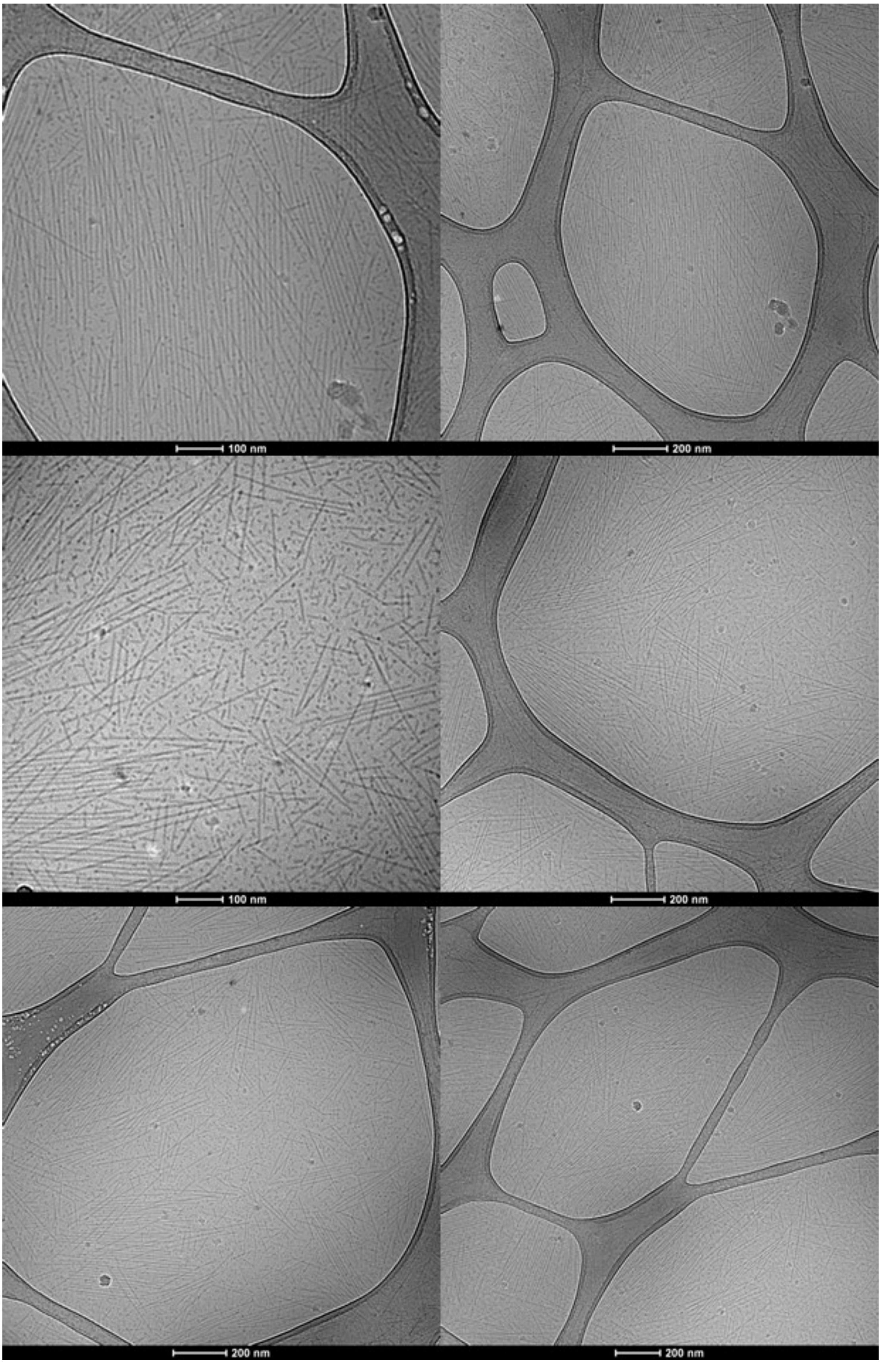

Figure S3.5 Cryogenic transmission electron micrograph (cryo-TEM) of **1** (c = 2 mM) with **4** (2 mol%) in water at various magnifications

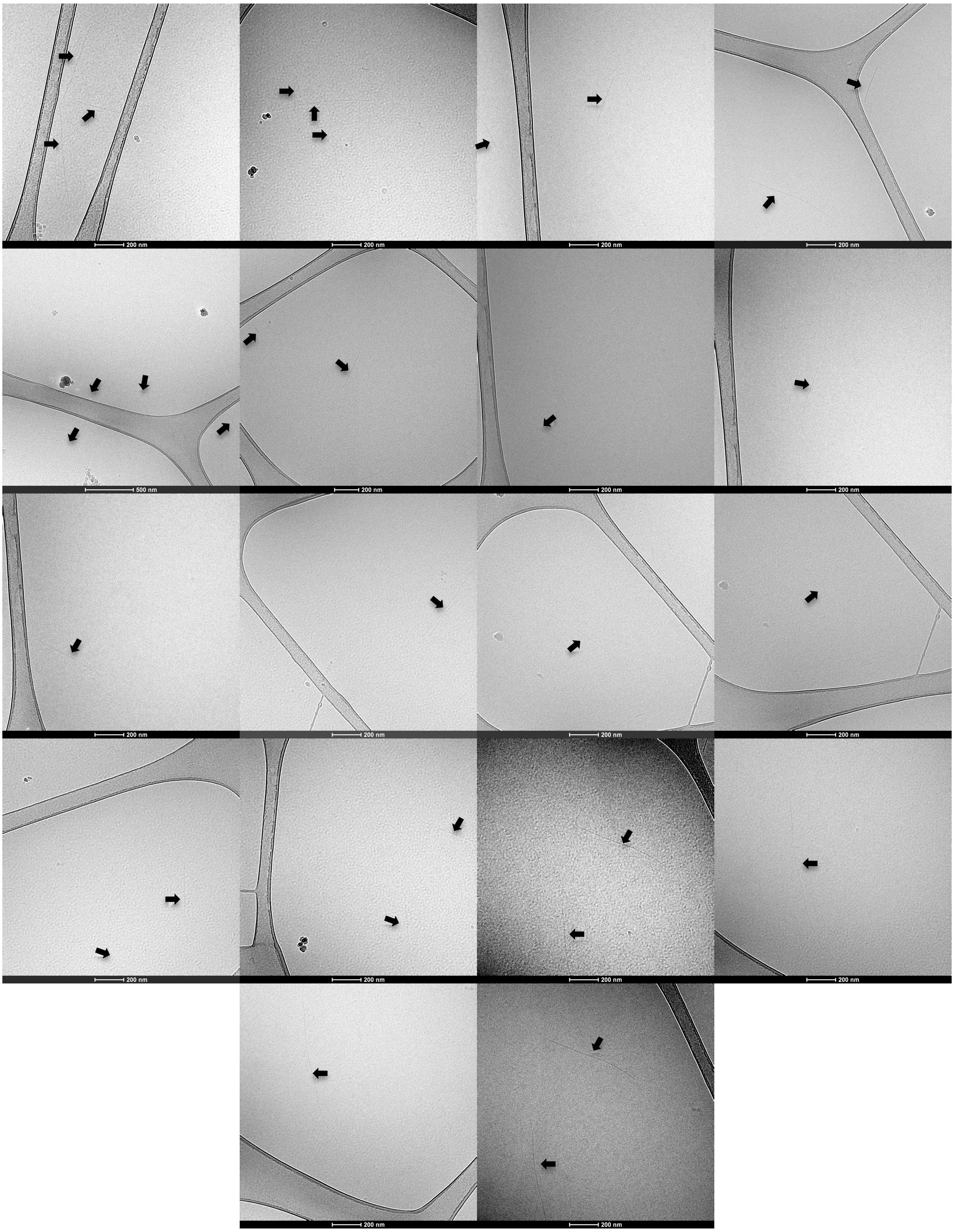

Figure S3.6 Representative cryogenic transmission electron micrographs (cryo-TEM) of **4** (c= 40 µM) in water. Monomer **4** forms fibrillar aggregates (black arrows indicate representative fibers) with a length of 387 ± 194 nm (N=20), however these structures are not observed in similar samples when monomer **4** is mixed with compounds **2** or **3,** suggesting monomer co-assembly occurs.

4. Critical aggregation concentration determination

DLS experiments to determine the critical aggregation concentrations were performed using a Zetasizer Nano-S (Malvern) across a range of monomer concentrations prepared in Milli-Q water. Samples were left to stand for 2 h prior to measurement.

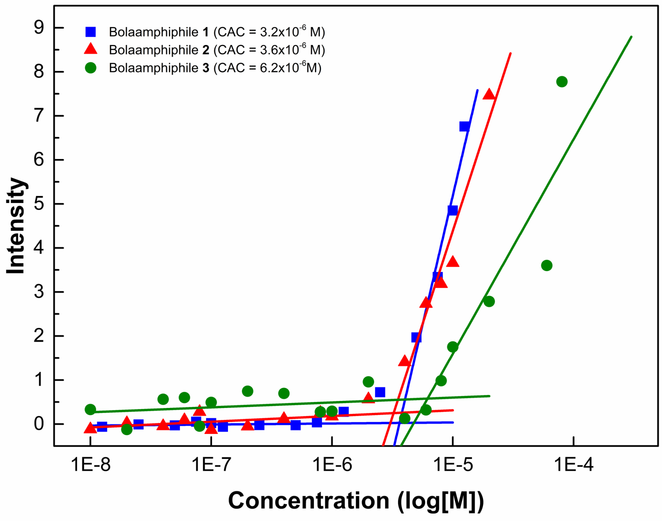

Figure S4.1 Scattered light intensity as a function of monomer concentration of **1**, **2**, and **3** to determine the critical aggregation concentration. The scattered light intensity is displayed as intensity relative to recorded background intensity of Milli-Q water.

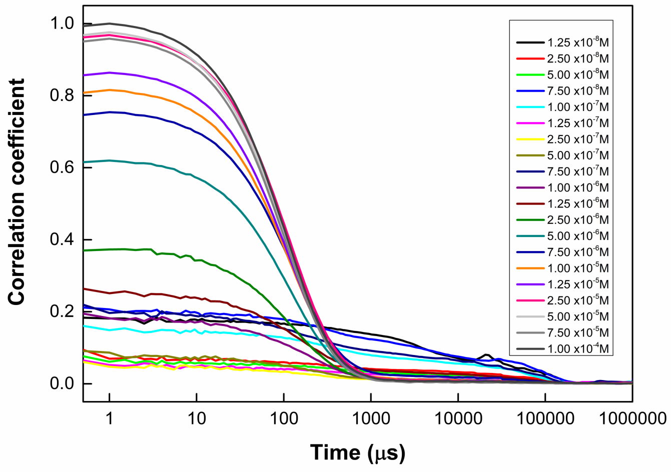

Figure S4.2 DLS correlograms of **1**.

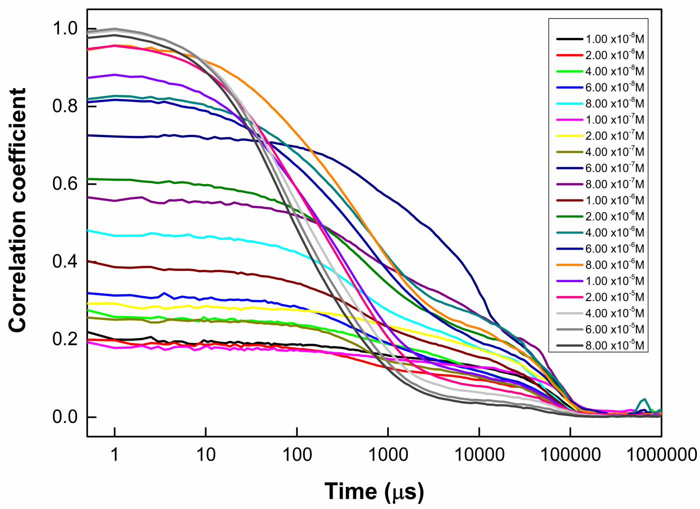

Figure S4.3 DLS correlograms of **2**.

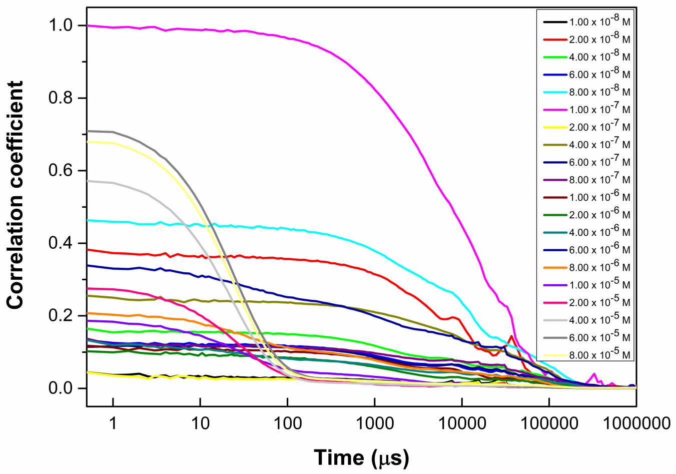

Figure S4.4 DLS correlograms of **3**.

5. UV-Vis spectra

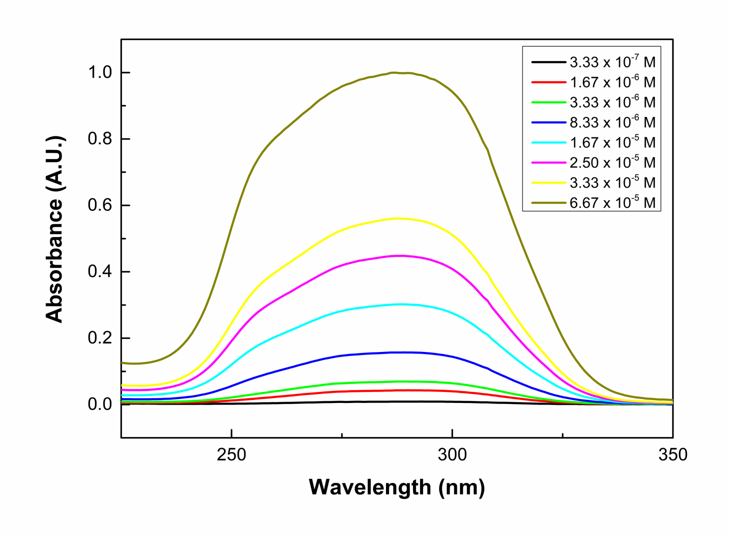

Figure S5.1 Normalized UV-Vis spectra (225-350 nm) of **3** above and below the critical aggregation concentration.

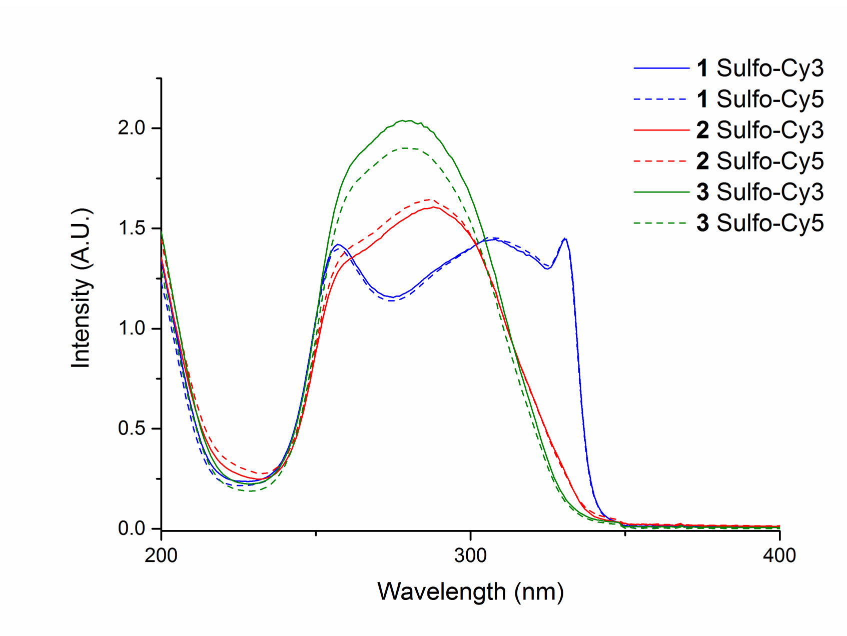

Figure S5.2 UV-Vis spectrum (200-400 nm) of **1**, **2** and **3** (c = 30µM) with 2 mol% **4** (solid line) and **5** (dashed line).

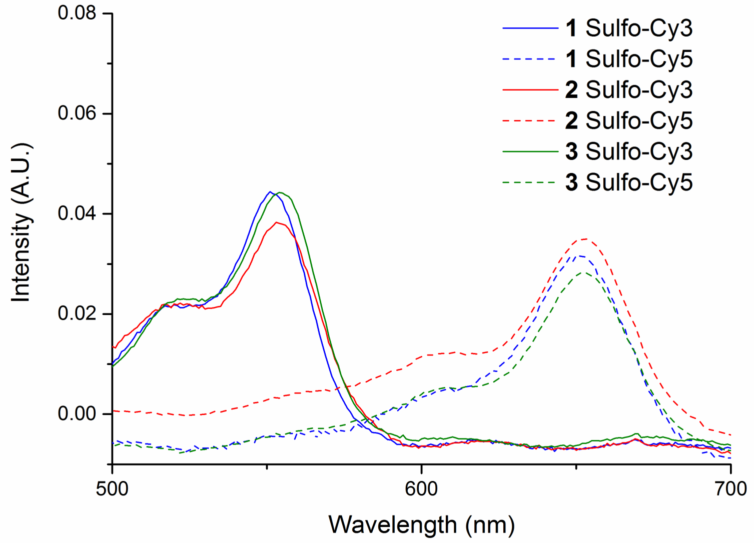

Figure S5.3 UV-Vis spectrum (500-700 nm) of **1**, **2** and **3** (c = 30 µM) with 2 mol% **4** (solid line) and **5** (dashed line).

6. Fluorescence spectra

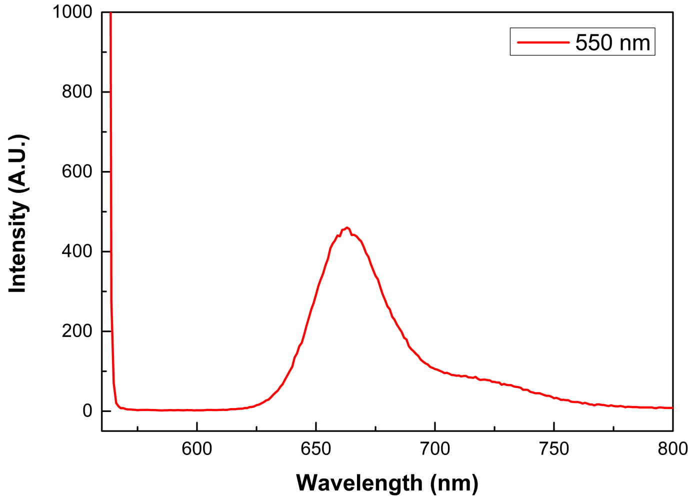

Figure S6.1 Fluorescence spectrum of free Sulfo-Cy5 dye (0.3 µM) (λ_{exc}= 550 nm λ_{em}= 670 nm).

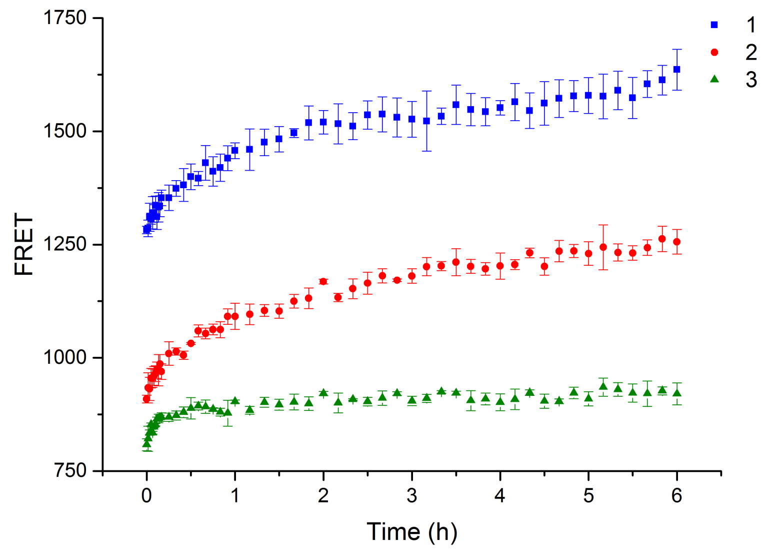

Figure S6.2 Raw fluorescent data (λ_{exc}= 550 nm λ_{em}= 670 nm) measuring the FRET channel in dynamic experiments where solutions of **1** (blue), **2** (red) and **3** (green) labeled with **4** or **5** (2 mol%) are mixed in an equimolar ratio at room temperature.

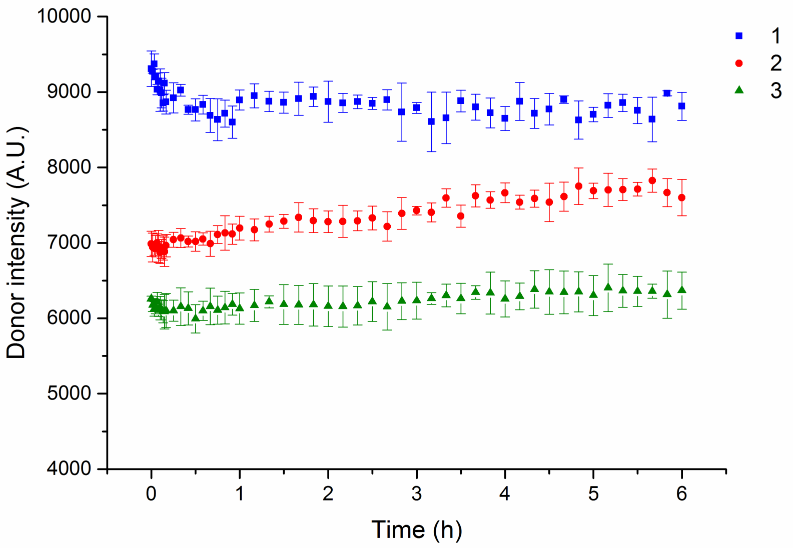

Figure S6.3 Raw fluorescence data from the donor (λ_{exc}= 550 nm λ_{em}= 570 nm) channel from FRET dynamic experiments where solutions containing **1** (blue), **2** (red) and **3** (green) labeled with **4** or **5** (2 mol%) are mixed in an equimolar ratio at room temperature.

7. Cryo-TEM of 1 with carp serum

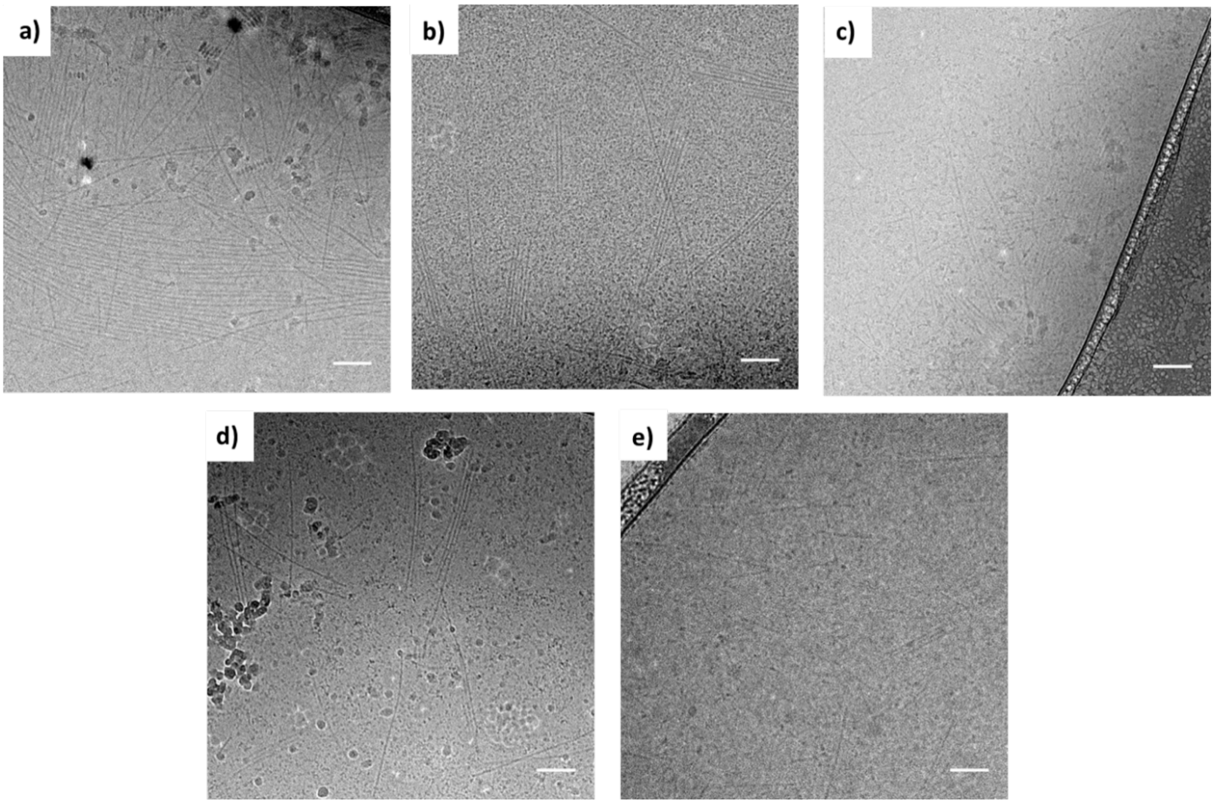

Figure S7.1 Cryo-TEM images of squaramide-based supramolecular polymer nanoparticles of **1** (2 mM) with 2 mol% **4** in a solution with increasing carp serum from 1-25 v/v%: 1% (a), 2.5% (b), 5% (c), 10% (d) and 25% (e). Fibrillar structures are retained with increasing carp serum concentration. The scale bar represents 100 nm.

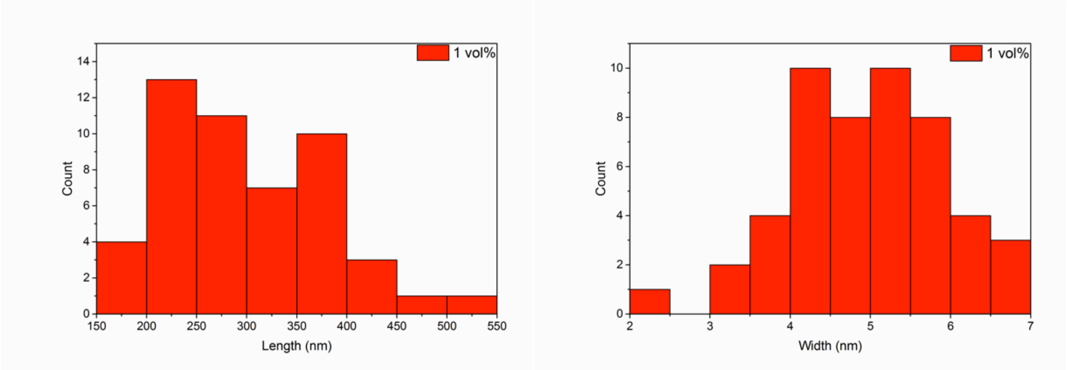

Figure S7.2 Histograms of length (299 ± 78 nm) and width (5 ± 1 nm) distributions measured for **1** at 1 v/v% of carp serum (n= 50) measured from Cryo-TEM images.

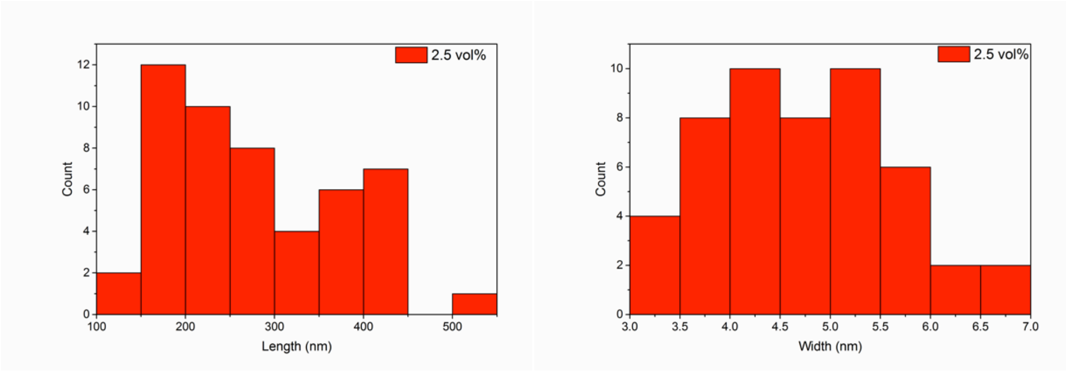

Figure S7.3 Histograms of length (277 ± 103 nm) and width (5 ± 1 nm) distributions measured for **1** at 2.5 v/v% of carp serum (n= 50) measured from Cryo-TEM images.

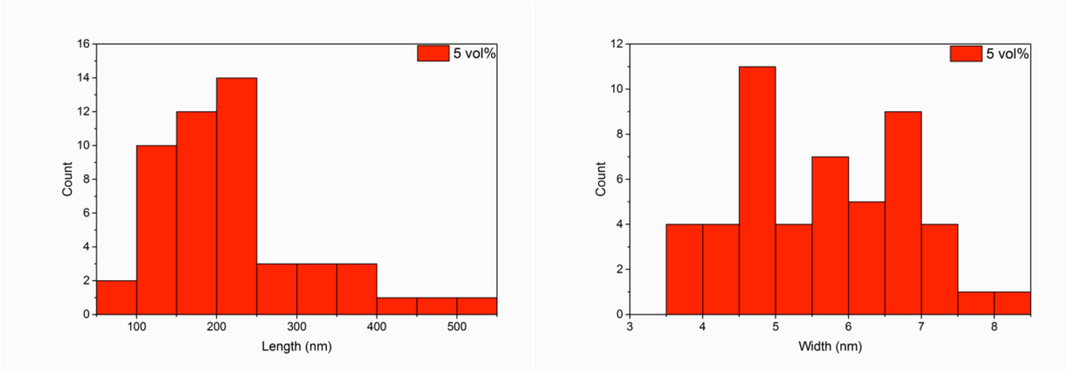

Figure S7.4 Histograms of length (218 ± 96 nm) and width (6 ± 1 nm) distributions measured for **1** at 5 v/v% of carp serum (n= 50) measured from Cryo-TEM images.

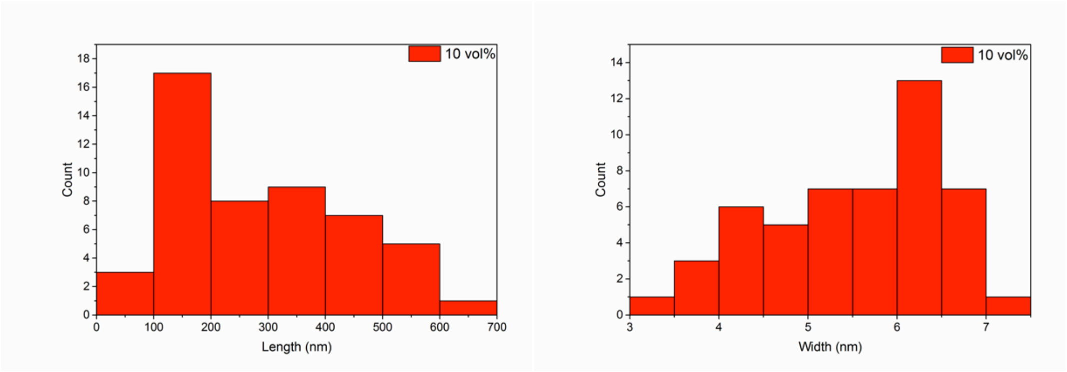

Figure S7.5 Histograms of length (288 ± 142 nm) and width (6 ± 1 nm) distributions measured for **1** at 10 v/v% of carp serum (n= 50) measured from Cryo-TEM images.

8. Zebrafish husbandry, injections and whole embryo imaging

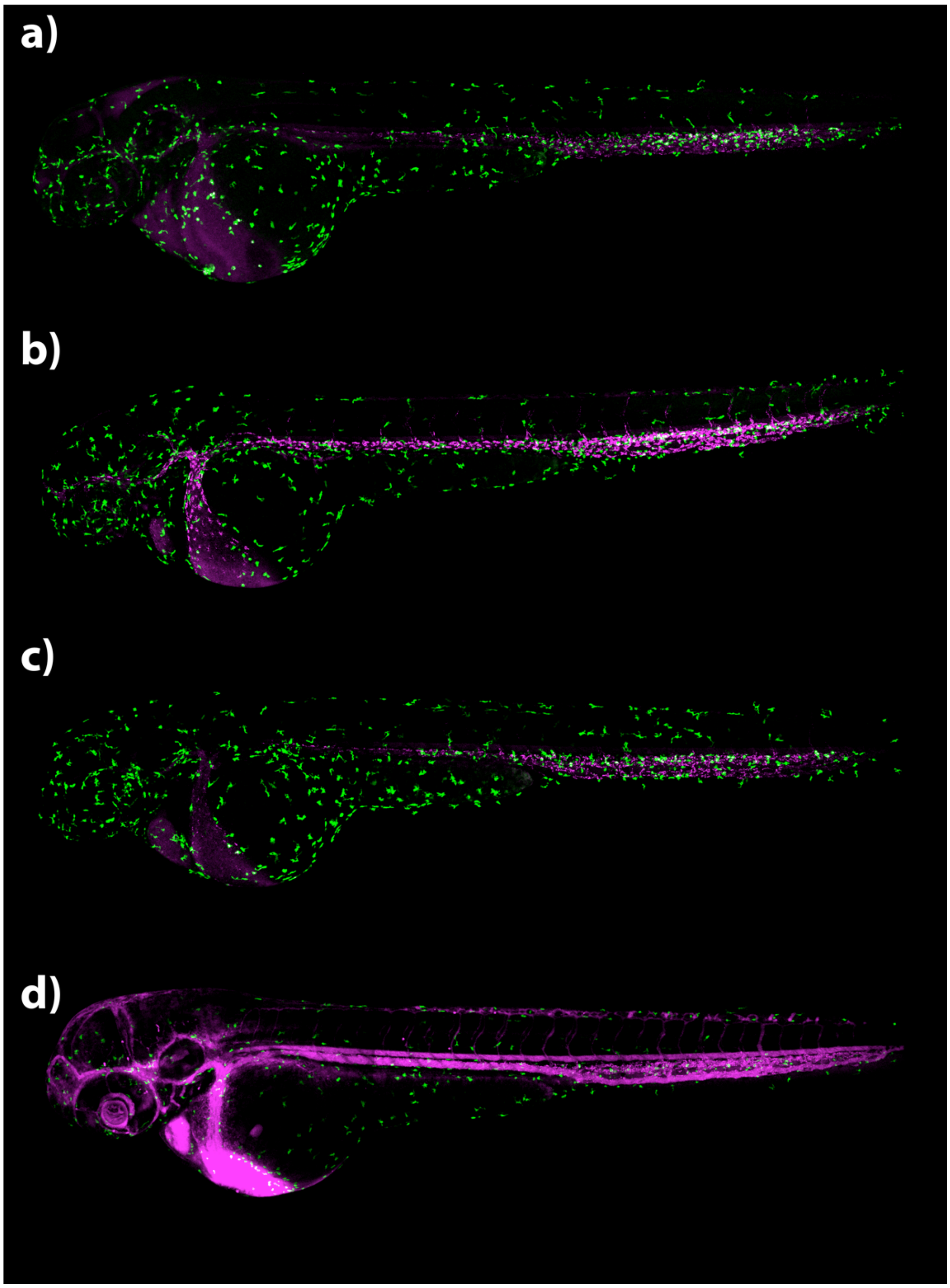

Figure S8.1 Uptake studies of the fluorescently-labeled squaramide-based supramolecular polymer nanoparticles by macrophages in the zebrafish. Whole-embryo view 1 hour post-injection in the Duct of Cuvier with **1** (a), **2** (b) and **3** (c) **1**, **2** or **3** (c = 2 mM) and 2 mol% of **4** in an embryonic zebrafish Tg(*mpeg1:GFP*) at 54 hpf. (d) Whole-embryo view after 1 hour injection in the Duct of Cuvier with **4** (c = 40 µM) on its own in an embryonic zebrafish at 54 hpf. This control experiment is carried out to track the uptake of the negatively charged particles of **4** by macrophages studies in zebrafish.

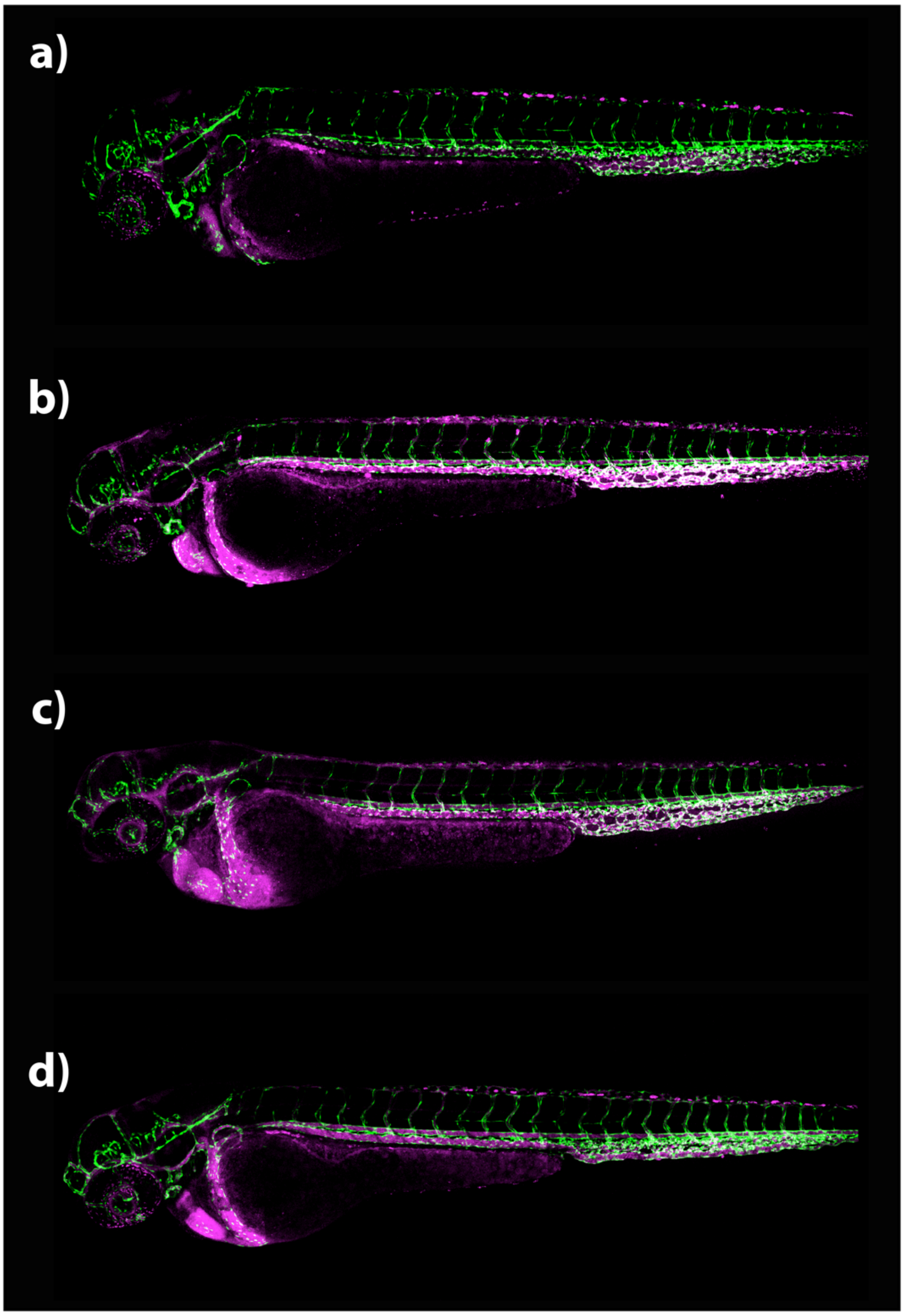

Figure S8.2 Biodistribution of the fluorescently-labeled squaramide-based supramolecular polymer nanoparticles in the zebrafish. Whole-embryo view 1 hour post-injection in the Duct of Cuvier with **1** (a), **2** (b) and **3** (c) **1**, **2** or **3** (c= 2 mM) co-assembled with 2 mol% **4** in an embryonic zebrafish *Tg(kdrl:GFP*) at 54 hpf. (d) Whole-embryo view after 1 hour injection in the Duct of Cuvier with **4** (c = 40 μM) on its own in an embryonic zebrafish *Tg*(*kdrl:GFP*) at 54 hpf. This control experiment is carried out to track the biodistribution of the negatively charged monomer itself.

9. References

1. Saez Talens, V.; Englebienne, P.; Trinh, T. T.; Noteborn, W. E.; Voets, I. K.; Kieltyka, R. E., Aromatic Gain in a Supramolecular Polymer. *Angew Chem Int Ed Engl* **2015,** *54* (36), 10502-6.
2. Saez Talens, V.; Makurat, D. M. M.; Liu, T.; Dai, W.; Guibert, C.; Noteborn, W. E. M.; Voets, I. K.; Kieltyka, R. E., Shape modulation of squaramide-based supramolecular polymer nanoparticles. *Polymer Chemistry* **2019,** *10* (23), 3146-3153.
3. Dardonville, C.; Fernandez-Fernandez, C.; Gibbons, S. L.; Ryan, G. J.; Jagerovic, N.; Gabilondo, A. M.; Meana, J. J.; Callado, L. F., Synthesis and pharmacological studies of new hybrid derivatives of fentanyl active at the μ-opioid receptor and I2-imidazoline binding sites. *Bioorg Med Chem* **2006,** *14* (19), 6570-80.
